# Cuproptosis in iron overload hepatocytes

**DOI:** 10.1038/s41419-026-09275-y

**Published:** 2026-09-19

**Authors:** Judith Sailer, Jonas Gottal, Leon Kaub, Adrian T. Jauch, Jonas Engler, Carola Eberhagen, Christine von Toerne, Lea Hansen-Palmus, Valerie Wachinger, Banu Akdogan, Stefan Engelhardt, Percy Knolle, Alan A. DiSpirito, Ulrike Protzer, Maja Vujić Spasić, Hans Zischka

**Affiliations:** 1 Institute of Toxicology and Environmental Hygiene, TUM School of Medicine and Health, Technical University of Munich, Munich, Germany; 2 Department of Computer Science, TUM School of Computation, Information and Technology, Technical University Munich (TUM), Garching, Germany; 3 Department of Earth and Environmental Sciences, LMU Munich, Munich, Germany; 4 Department of Anatomy II, Faculty of Medicine, LMU Munich, Munich, Germany; 5 Institute of Molecular Toxicology and Pharmacology, Helmholtz Munich, German Research Center for Environmental Health, Neuherberg, Germany; 6 Metabolomics and Proteomics Core, Helmholtz Center Munich, German Research Center for Environmental Health, Munich, Germany; 7 Institute of Virology, School of Medicine, Technical University of Munich, Munich, Germany; 8 Institute of Virology, Helmholtz Munich, German Research Center for Environmental Health, Neuherberg, Germany; 9 Institute of Pharmacology and Toxicology, TUM School of Medicine and Health, Technical University of Munich, Munich, Germany; 10 Institute of Molecular Immunology, Technical University of Munich (TUM), School of Medicine and Health, Munich, Germany; 11 Roy J. Carver Department of Biochemistry, Biophysics and Molecular Biology, Iowa State University, Ames, Iowa USA; 12 German Center for Infection Research (DZIF), Munich, Germany; 13 Institute of Comparative Molecular Endocrinology, Ulm University, Ulm, Germany

**Keywords:** Metabolic disorders, Cell death

## Abstract

A long-standing clinical conundrum is the pronounced phenotypic heterogeneity of hereditary hemochromatosis. Patients with identical *HFE* genotypes and similar degrees of iron overload can have vastly different clinical outcomes, ranging from being completely asymptomatic to developing severe cirrhosis, diabetes, or cardiomyopathy. This variability strongly implies the existence of genetic or environmental modifiers that dictate disease penetrance. Here, copper may be a compelling disease-modifying candidate, due to its intimately intertwined metabolism with iron. Indeed, profound iron loading in hemochromatosis mice (*Hfe* knockout) and hemochromatosis Huh7 hepatocytes does not cause overt toxicity. However, the addition of copper, even in trace amounts and harmless on its own, dramatically amplifies adverse effects to induce profound cell toxicity. We find that copper-provoked iron vulnerability is not driven by Fenton chemistry-driven ROS damage, but instead induces a cuproptotic-like mechanism, characterized by proteotoxic stress, loss of iron-sulfur cluster enzyme activity, and severe mitochondrial dysfunction. Remarkably, high-affinity copper chelation rescued Huh7 cells, whereas inhibition of other canonical cell death pathways did not. These findings spotlight copper as a critical modifier in iron overload toxicity, capable of converting largely compensated iron overload into severe cellular injury and death, thereby providing a mechanistic explanation for the phenotypic heterogeneity in hemochromatosis.

**Figure:**
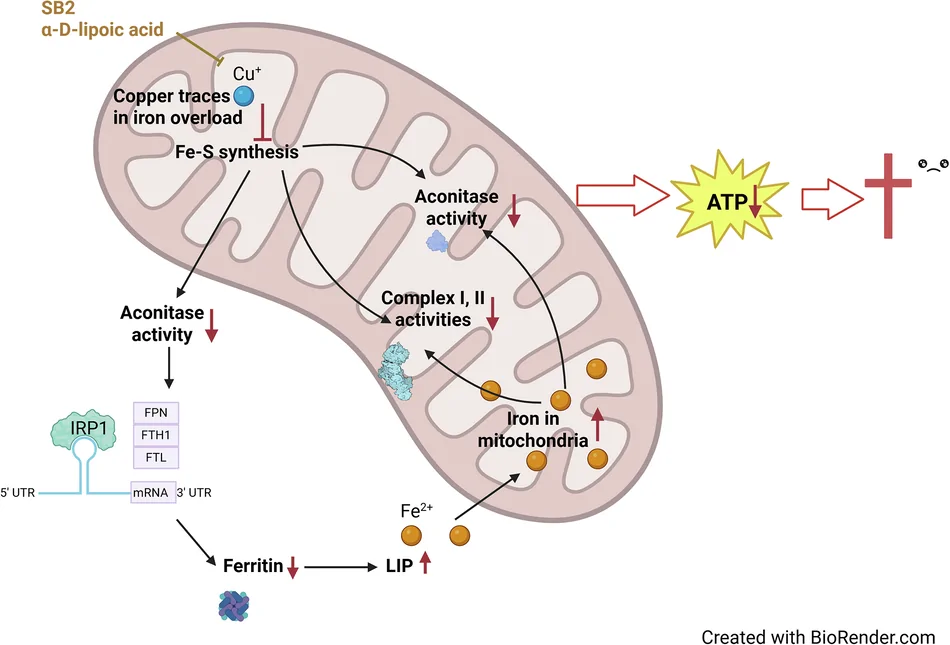

## Introduction

Iron (Fe) is essential for oxygen transport, cellular respiration, and DNA synthesis, but its redox cycling between Fe^2+^ and Fe^3+^ can generate deleterious reactive oxygen species (ROS) via Fenton chemistry [1–4].

Our strict health dependency on sufficient iron is maybe best exemplified by the lack of a physiological Fe excretion route [5]. However, this absence is of profound toxicological concern in situations of increased systemic iron supply, such as with repeated blood transfusions [6]. While we may lose iron, e.g., by cell desquamation or menstruation, systemic Fe-turnover is primarily regulated via the hepcidin-ferroportin regulatory axis. Hepcidin, a liver-derived hormone, reduces the export of nutritional iron from the intestine into the blood via interaction with the Fe-exporter ferroportin [7–10]. Consequently, genetic conditions that affect this regulatory axis cause iron overload, collectively described as hereditary hemochromatosis (HH) [11].

HH, due to mutations in the *HFE* gene, is the most prevalent genetic iron overload disease in northern Europe, with an incidence of 1/200 to 1/400 [12]. The most common C282Y mutation in the *HFE* gene causes hepcidin deficiency and subsequent increased iron release from the GI-tract via ferroportin into blood [9, 11], with iron being deposited primarily in liver, heart, and pancreas [11, 13]. A particular conundrum in hemochromatosis is its incomplete penetrance, causing clinical manifestations, e.g., in only 5-29% of individuals who are homozygous for the C282Y mutation [14, 15]. While many affected individuals remain asymptomatic throughout life, others may develop severe complications, including acute liver failure, cirrhosis, hepatocellular carcinoma, arthropathy, cardiomyopathy, and diabetes [11].

Incomplete penetrance typically is due to individual factors (e.g., age, sex, genetic peculiarities) or environmental/nutritional aspects; it may, however, also be due to possible confounding factors [16]. Here, copper emerges as a compelling modifier of iron overload phenotypes due to its biochemical interconnection with iron metabolism, comparable redox cycling capacity, and partially shared transport systems, including DMT1 [17]. Both metals are absorbed intestinally and stored primarily in the liver [18]. Paradoxically, iron overload can accompany copper deficiency [19], while copper deficiency causes secondary iron accumulation [20], plausibly due to impaired ceruloplasmin and hephaestin function [19, 21, 22]. Conversely, hepatic copper accumulation has been documented in iron overload disorders [23], suggesting complex bidirectional interactions.

Driven by these findings, we examined whether copper influences cellular stress during iron overload. Iron excess in livers of *Hfe* knockout mice, the standard model of hereditary hemochromatosis [24, 25], and in *HFE*-deficient Huh7 cells [26] triggered adaptive lysosomal changes without overt toxicity. However, addition of trace copper, otherwise harmless, provoked severe cell death via copper-iron proteotoxicity that impairs iron-sulfur enzymes and causes mitochondrial dysfunction, indicating a copper-induced, iron-dependent bioenergetic crisis. Notably, while inhibitors of apoptosis, necroptosis, ferroptosis, or iron toxicity were largely ineffective, potent copper chelation fully prevented cell death. Thus, upon iron overload, even trace amounts of copper can be devastating. These findings suggest that a cuproptosis-like cell death may be a critical determinant in hepatic iron overload disease.

## Results

### Iron overload in hemochromatosis mice hardly causes liver toxicity

**Fig. 1:**
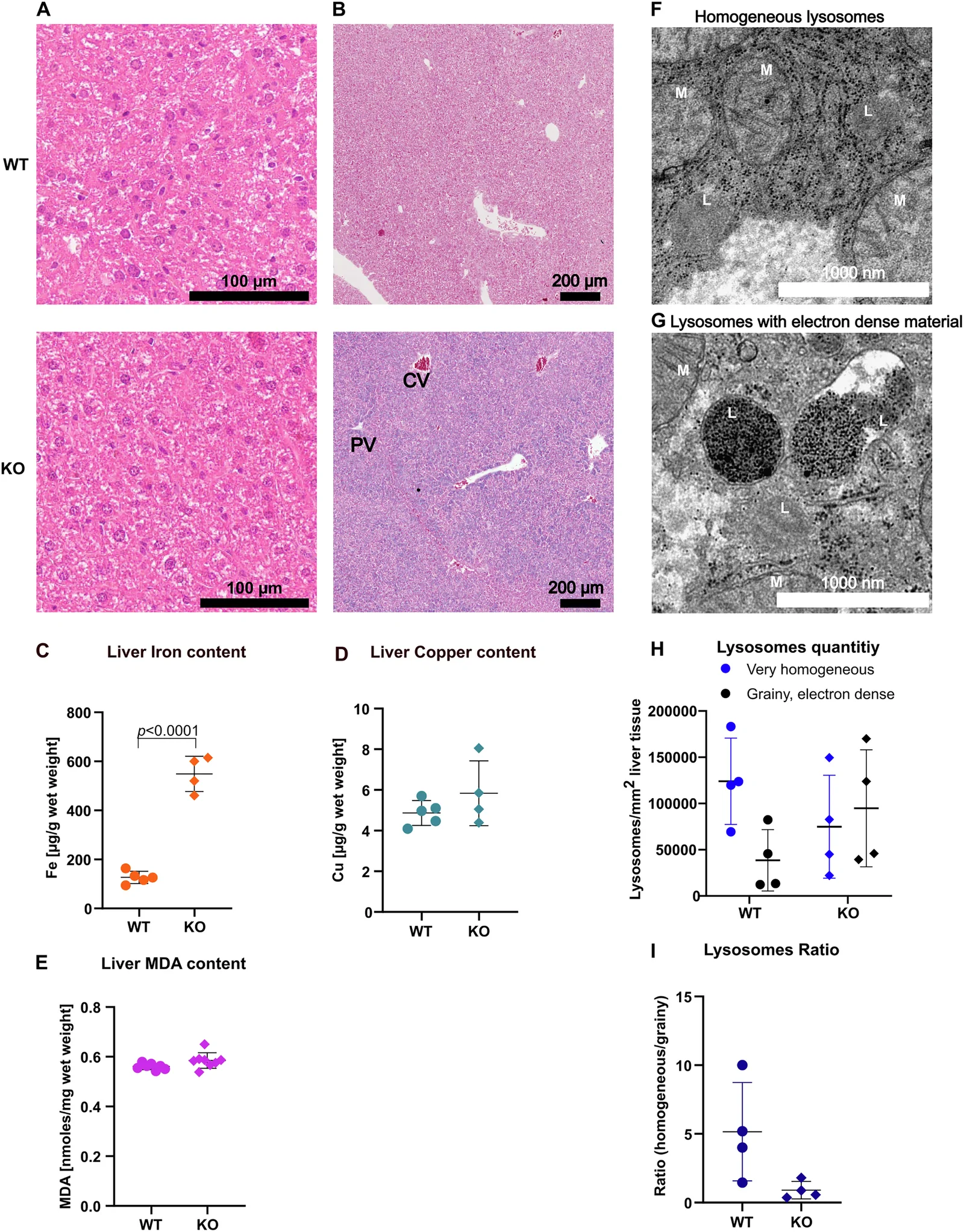
Hepatic iron accumulation in Hfe KO mice does not provoke overt pathological alterations. **A** Hematoxylin and eosin (H&E) staining of liver sections from age-matched (15-16 weeks of age) female *Hfe* wild type (WT) and KO mice. **B** Perls’ Blue staining of liver sections from these mice, with PV indicating portal vein and CV central vein. **C** Liver iron and **D** copper concentration measured by ICP-OES in *Hfe* WT (N = 5) and KO (N = 4) mice aged 14-18 weeks of age. **E** Malondialdehyde (MDA) levels in liver tissue of *Hfe* WT (N = 7) and KO (N = 8) mice at an age of 17 weeks. Transmission electron micrographs of liver sections from *Hfe* WT and KO mice without morphological alterations of mitochondria but depicting distinct lysosomal subtypes with **M**: mitochondria; **L**: lysosomes: (**F**) *Hfe* WT mice with homogeneous lysosomes and (**G**) *Hfe* KO mice with a higher abundance of lysosomes containing grainy, electron-dense material. **H** Quantitative analysis of lysosomal populations in liver samples from WT (*N* = 4) and KO (*N* = 4) mice using ImageJ. **I** The ratio of homogeneous to grainy lysosomes is plotted for age-matched *Hfe* WT and KO mice (*N* = 4 per group). An unpaired t-test was employed for statistical evaluation of (**C**–**E**) and (**I**) and one-way ANOVA was employed in (**H**), followed by Tukey’s post hoc test for multiple comparisons. The horizontal line shows the mean, with the error bars representing the standard deviation.

Hemochromatosis *Hfe* KO mice have a hepatic iron deposition pattern that perfectly resembles the situation in human disease [27, 28]. In agreement with a majority of hemochromatosis patients [15, 29–31], studies do not report overt signs of liver damage in these mice [27, 32, 33]. Indeed, H&E staining of liver tissues from *Hfe*^+/+^ (WT) and *Hfe*^-/-^ (KO) mice were highly comparable healthy, and the latter did not show notable histological alterations (Fig. 1A). This was despite profound hepatic iron deposition in the *Hfe* KO mice, as evidenced by the characteristic blue discoloration on Perls’ blue staining (Fig. 1B), consistent with previous reports [24]. In line with this, the iron overload was further validated by quantitative measurement of hepatic iron content using ICP-OES. Here, *Hfe* KO mice had a significant increase in liver iron concentration vs. *Hfe* WT animals (*p* < 0.0001, Fig. 1C), whereas copper levels were low and did not differ between the genotypes (Fig. 1D). Despite this high iron loading, there was no sign of increased oxidative damage in the liver of KO animals compared to wild types, as evidenced by equal levels of lipid peroxidation (Fig.1E).

At ultrastructural level, *Hfe* KO mouse livers appeared like WT controls (Fig. 1F, G), e.g., showing intact and well-structured mitochondria (Fig. 1F, G). Interestingly, however, a visibly increased number of lysosomes with characteristic grainy, electron-dense material were apparent in hemochromatosis mice. This became more apparent by determining a ratio of homogeneous lysosomes vs grainy lysosomes (Fig. 1H, I). This indicates alterations in the lysosomal system in the iron-loaded mice. As excess iron is typically stored in cytosolic ferritin [5, 8] to be mobilized via autophagy in lysosomes (then called “ferritinophagy” [34]), this finding may plausibly indicate an elevated iron turnover in these animals (see below). Notably, the reported diameter of the ferritin iron core (~8 nm, [35]), as well as that of formed iron nanoparticles [36], agrees well with the size of such electron-dense granules observed in the grainy liver lysosomes of the *Hfe* KO mice (Fig. 1G).

### Human hemochromatosis Huh7 cells are resistant to high ferric ammonium citrate challenges

**Fig. 2:**
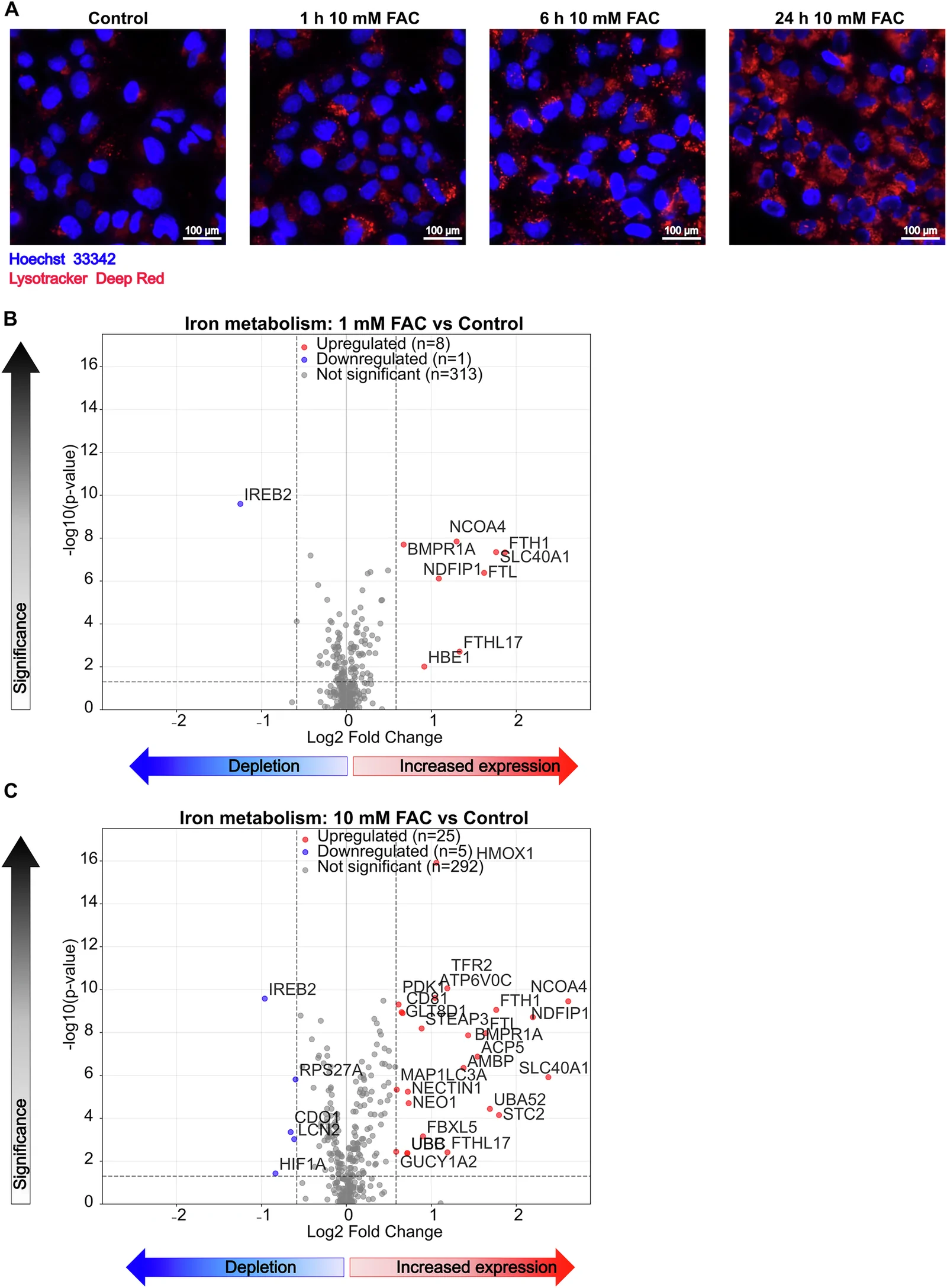
Iron treatment of Huh7 cells strongly increases lysosomal abundance and provokes changes in iron handling. **A** Fluorescence micrographs of Huh7 cells treated with 10 mM FAC for 1, 6, or 24 hours, vs. left untreated (control), stained with LysoTracker™ Deep Red (red) and Hoechst 33342 (blue). Image overlays were generated using ImageJ. Changes in the proteome of Huh7 cells treated with (**B**) 1 mM or (**C**) 10 mM FAC for 24 hours compared to untreated Huh7 cells, filtered for proteins involved in iron metabolism (*N* = 4), are depicted as a volcano plot. Data points represent log2 fold change (x-axis) versus -log10(p-value) (y-axis). Dotted lines indicate significance thresholds (*p* < 0.05) and fold change >1.5.

We next exposed Huh7 cells, a model of human hemochromatosis hepatocytes [26], to high concentrations of ferric ammonium citrate (FAC) for 1, 6 and 24 hours to investigate the effect of high iron loading on the lysosomal system. FAC increased the lysosomal content significantly in a time-dependent manner, evidenced by LysoTracker™ Deep Red staining (Fig. 2A, SFig. S1A 1 h to control: *p* = 0.0007; 4 h to control: *p* < 0.0001; 24 h to control: *p* < 0.0001). As in vivo lysosomes with grainy electron-dense content may be indicative of increased ferritinophagy (Fig. 1G), we then analyzed changes in the proteome of these cells upon iron treatment and sorted for proteins involved in iron metabolism. A clear elevation of ferritin (FTH1 and FTL), Nuclear Receptor Coactivator 4 (NCOA4), which mediates ferritinophagy [34], as well as ferroportin (SLC40A1), the cellular iron exporter, was already observed upon treatment with 1 mM FAC for 24 h (Fig. 2B), which increased profoundly in significance upon treatment with 10 mM FAC (Fig. 2C). NCOA4 reproducibly increased as well, already upon 1 h FAC exposure (SFig. S1B), and a notable dose-dependent abundance elevation was also found for ferritin (SFig. S1C). Thus, as observed in vivo, Huh7 cells clearly aim to balance the imposed profound iron challenge by increased ferritin storage, but also by its mobilization via ferritinophagy and excretion via ferroportin.

### Copper traces elicit iron toxicity in hemochromatosis hepatocyte surrogate cells

**Fig. 3:**
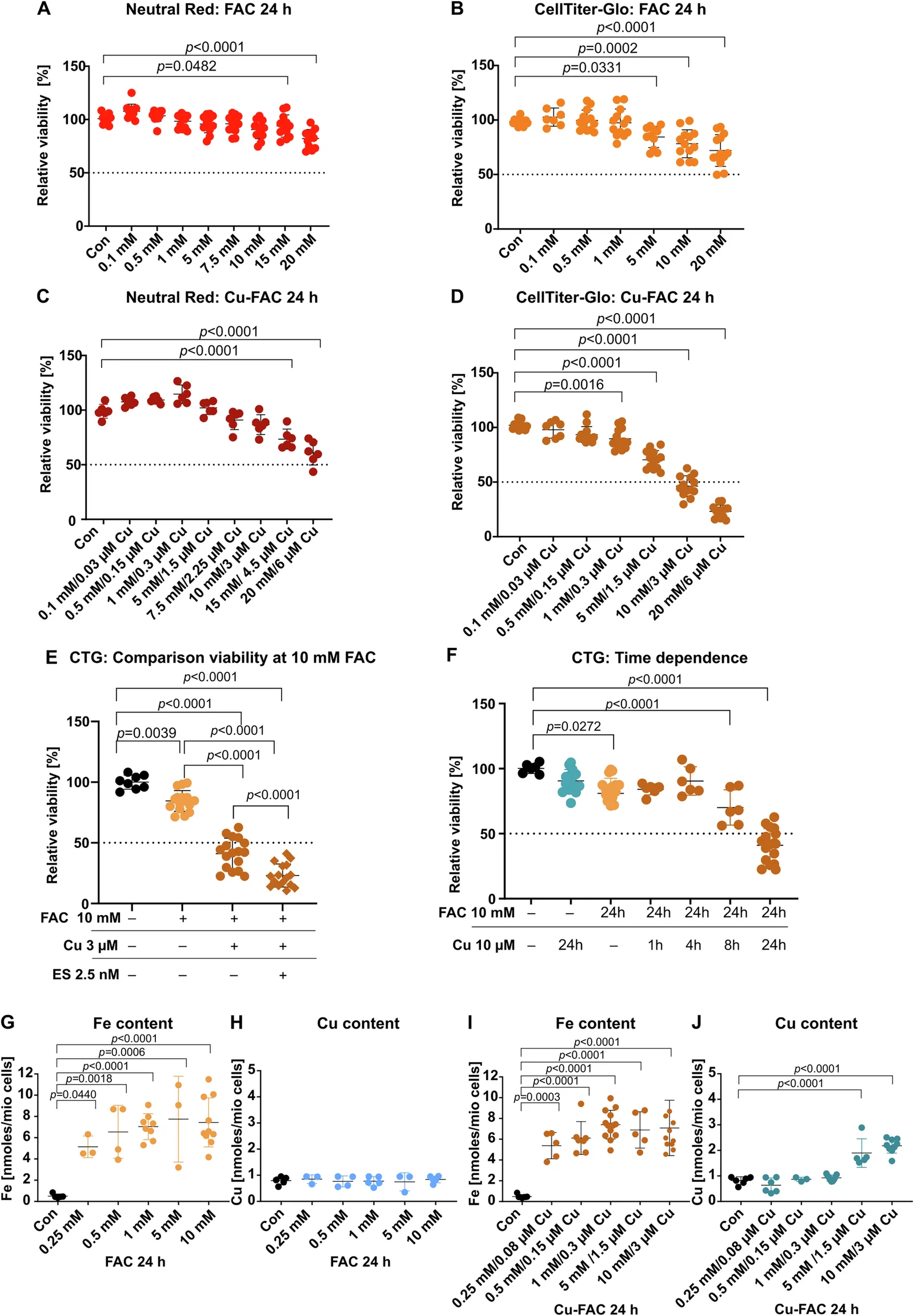
Copper traces massively enhance iron-induced cytotoxicity in Huh7 cells. **A** Neutral Red uptake assay of Huh7 cells treated for 24 hours with increasing concentrations of ferric ammonium citrate (FAC). Data are expressed as percentage vs. untreated controls (*N* = 6; *n* = 2). **B** Cell viability measured by CellTiter-Glo® (CTG) after 24 hours treatment with increasing concentrations of FAC. Data are expressed as a percentage vs. untreated controls (*N* ≥ 3, *n* = 2, 3). **C** Neutral Red uptake assay following 24 hours treatment with copper-containing FAC (FAC stock supplemented with 30 µM Cu in 100 mM FAC molar ratio 1:3333) at increasing concentrations. Data are expressed as percentage vs. untreated controls (*N* = 3, *n* = 2). **D** Viability determined via CTG after 24 hours treatment with copper-containing FAC at increasing concentrations (*N* ≥ 3, *n* = 2, 3). **E** Comparison of the cell viability of Huh7 cells treated with 10 mM with or without copper and 2.5 nM elesclomol (ES) or left untreated, determined via CTG (N ≥ 8). **F** Time-course viability of cells treated with 10 µM copper, 10 mM FAC, or both for the indicated times (*N* ≥ 4, *n* = 2) determined via CTG. Intracellular iron (**F**) and copper (**G**) content was measured by ICP-OES following 24 hours treatment with FAC or copper-containing FAC (*N* ≥ 3, *n* = 1). For statistical evaluation, one-way ANOVA was employed, followed by Tukey’s post hoc test for multiple comparisons. The horizontal line shows the mean, with the error bars representing the standard deviation.

We next exposed Huh7 cells to increasing concentrations of ferric ammonium citrate (FAC, from 0.1 to 20 mM) for 24 hours (Fig. 3A). As reported, toxicity thresholds vary widely across different studies [37, 38], and found that cells tolerated even millimolar levels of iron. Here we used the Neutral Red uptake assay to test for toxicity by assessing lysosomal integrity, which was well retained up to FAC concentrations of 10 mM. Mild toxicity was encountered at 15–20 mM FAC (15 mM: *p* = 0.0482; 20 mM: *p* < 0.0001), with remaining viability above 80% vs. control levels (Fig. 3A), indicating that the Huh7 cells balance such (non-physiologically) high iron challenges, plausibly by the enhanced mechanisms described above. This absence of overt iron toxicity was validated by using a further cell toxicity assay (CellTiter-Glo®, CTG) that monitors cellular ATP [39]. Here, FAC alone provoked mild toxicity from 5 mM upwards (*p* = 0.0331), indicating mild bioenergetic cell stress by iron excess, but cell viability remained around 70% vs. control levels, even at the highest tested FAC dose (Fig. 3B).

Cell viability, however, declined profoundly upon addition of trace copper to the FAC challenge (Fig. 3C–E; 100 mM FAC stock containing 30 μM Cu, molar ratio 3 333:1). In the Neutral Red uptake assay, viability started to decline at 5 mM FAC (1.5 μM Cu, non-significant) and then decreased further in a dose-dependent manner (cf. Fig. 3A vs C, 15 mM to control: *p* < 0.0001, 20 mM to control: *p* < 0.0001). Effects on cellular ATP were strongly aggravated in the presence of trace copper: while 20 mM FAC alone reduced viability to about 70% after 24 hours (*p* < 0.0001), the same FAC concentration containing 6 μM Cu decreased Huh7 cell viability to approximately 20% (cf. Fig. 3B vs D, *p* < 0.0001). Importantly, such copper traces alone, i.e., in the absence of iron overload, did not affect cellular viability (Fig. 3F, SFig. 2A).

To further demonstrate that this co-toxicity is driven/initiated by copper traces, we employed nanomolar concentrations of elesclomol, the paradigm inducer of cuproptosis that facilitates cellular copper entry [40, 41]. While elesclomol combined with copper caused some toxicity as well (SFig. S2A, D, 50% viability, compared to control *p* < 0.0001), Cu-FAC toxicity was devastating, completely eradicating the challenged Huh7 cells (Fig. 3E, SFig. S2D). Intriguingly, this Cu-initiated FAC toxicity occurred with a delay, becoming apparent only after several hours of incubation (Figs. 3, 8 h to control: *p* < 0.0001; 24 h to control: *p* < 0.0001). Moreover, while FAC alone progressively increased the cellular iron content to plateau at 5 mM, Cu levels stayed low (Fig. 3G, H). In contrast, when copper-traces were present, identical iron levels were reached, but also a significant Cu elevation was observed at higher dosing (Fig. 3I, J; 5 mM FAC/1.5 µM Cu: *p* < 0.0001; 10 mM FAC/3 µM Cu: *p* < 0.0001). Thus, it is this additional Cu that initiates the cellular toxicity, as the cellular iron burden was not affected (cf. Figure 3G vs. I).

### Copper-dependent iron toxicity may not be due to Fenton reactivity but due to protein toxicity

**Fig. 4:**
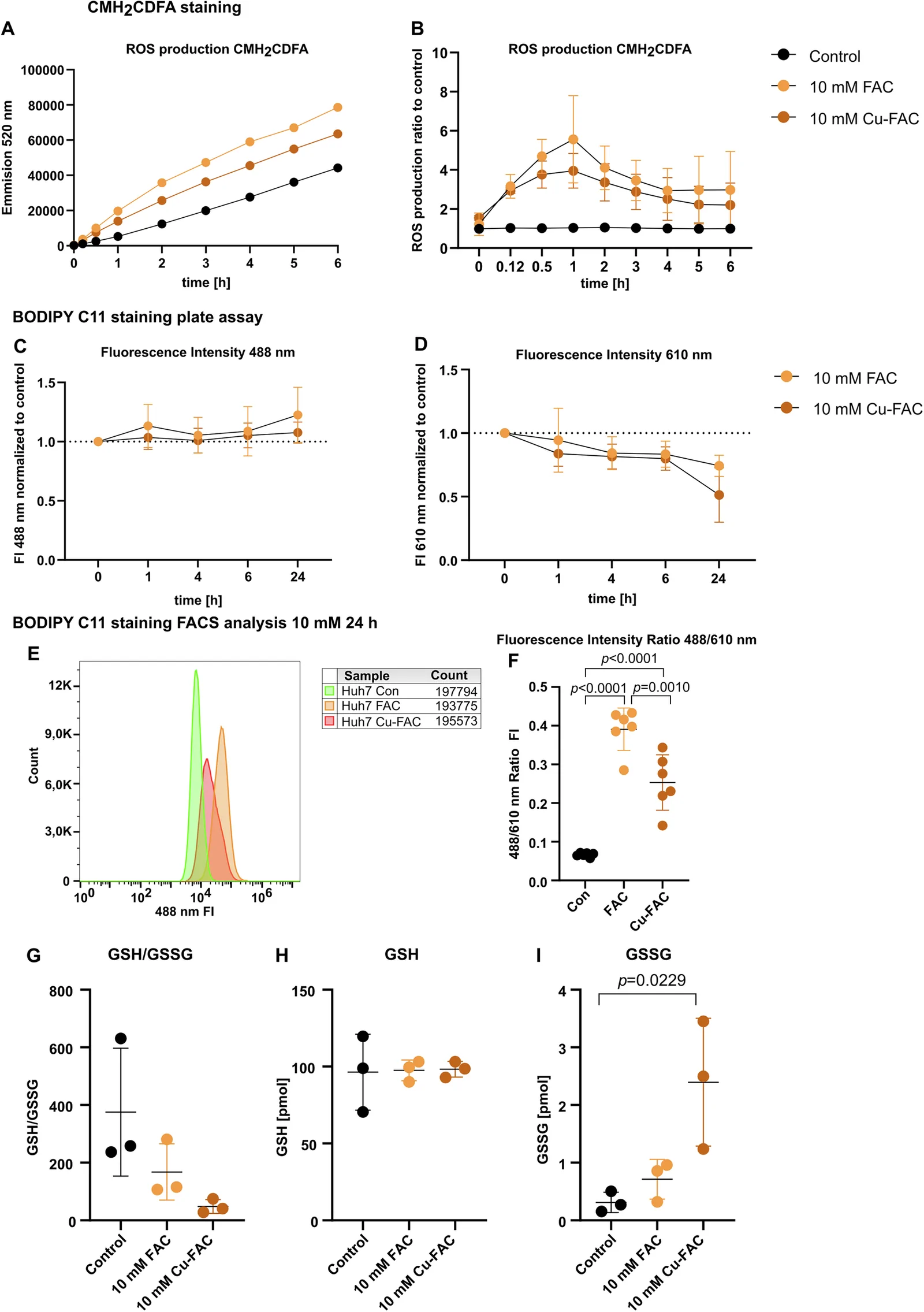
Copper-driven iron toxicity occurs independently of reactive oxygen species and lipid peroxidation. **A**, **B** Reactive oxygen species (ROS) production assessed using CM-H_2_DCFDA in Huh7 cells treated with 10 mM FAC or Cu-FAC up to 6 hours, normalized to untreated control (*N* = 3, *n* = 1). Lipid peroxidation assessed via BODIPY™ 581/591 C11 staining, (**C**) fluorescence intensity at 488 nm emission and (**D**) fluorescence intensity at 610 nm emission measured at the indicated time points upon 10 mM FAC or FAC-Cu treatment (*N* = 3, *n* = 1). **E** BODIPY C11 staining of Huh7 cells treated with 10 mM FAC or Cu-FAC for 24 hours, or untreated (Con), and analyzed via FACS for fluorescence emission at 488 nm, shown as a histogram. **F** Ratio of 488/610 nm fluorescence intensity (*N* = 4, *n* = 1). For statistical evaluation, one-way ANOVA was employed, followed by Tukey’s post hoc test for multiple comparisons. The horizontal line shows the mean, with the error bars representing the standard deviation. Glutathione assay determining total GSH (**H**), oxidized GSSG (**I**), and GSH/GSSG ratio (**G**) in cells treated for 24 hours with 10 mM FAC or copper-containing FAC (*N* = 3, *n* = 1). For statistical evaluation, one-way ANOVA was employed, followed by Tukey’s post hoc test for multiple comparisons. The horizontal line shows the mean, with the error bars representing the standard deviation.

The delayed onset of Cu-induced iron toxicity (Fig. 3F) casts transition metal-induced oxidative stress (via Fenton chemistry as a toxic mechanism) into doubt. Such reactivity would rather be expected to occur immediately upon incubation, giving rise to highly reactive hydroxyl radicals, lipid peroxidation, membrane damage, etc., i.e., processes collectively implicated in ferroptosis [2, 42]. Indeed, 10 mM FAC (w/o Cu traces) did provoke a brief rise in cellular ROS, as shown by increased CM-H_2_CDFA fluorescence (Fig. 4A). However, this was balanced out after one hour of incubation (Fig. 4B), in agreement with the absence of profound cell toxicity for FAC alone (Fig. 3A, C). Moreover, lipid peroxidation, as evidenced by BODIPY C11 staining, was hardly detectable by increased 488 nm nor by decreased 610 nm fluorescence in the plate assay (Fig. 4C, D), comparable to our in vivo observations of similar MDA liver content of *Hfe* KO and WT animals (Fig. 1E). The slightly lower 610 nm signal observed for 10 mM Cu-FAC after 24 h of incubation (Fig. 4D) may possibly be attributable to a toxicity-related reduction in cell number rather than being due to enhanced lipid peroxidation. Indeed, flow cytometric analysis confirmed this: a strong increase in the 488 nm fluorescence signal in FAC-treated cells was observed, which was weaker in Cu-FAC-treated cells (Fig. 4E). This difference became even more evident when analyzing the 488/610 nm fluorescence intensity ratio, which increased markedly upon FAC treatment (*p* < 0.0001 vs. control) but was significantly less pronounced in Cu-FAC-treated cells (*p* = 0.0011, FAC vs. Cu-FAC; Fig. 4F).

Contrary to Fenton-chemistry-based transition metal toxicity, especially copper toxicity has been more recently attributed to direct attack on thiol residues in vulnerable - mostly mitochondrial - proteins [17, 43]. Indeed, 10 mM FAC slightly, but 10 mM Cu-FAC strongly decreased the GSH/GSSG ratio, essentially due to GSSG formation (Fig. 4E–G; 10 mM Cu-FAC to control *p* = 0.0229). Such formation would be expected to counterbalance enhanced protein modifications, e.g., protein disulfide bond formation or S-glutathionylation [44, 45]. Furthermore, our proteome analysis revealed an induction of stress-induced heat shock protein HSP70 in Cu-FAC-treated cells (Supplementary Fig. S8C). It thus appears that the toxicity of Cu-FAC may rather be due to proteotoxic mechanisms than due to uncontrolled Fenton-based oxidative stress.

### Copper-driven iron toxicity can be ameliorated by thiol-containing copper chelators

**Fig. 5:**
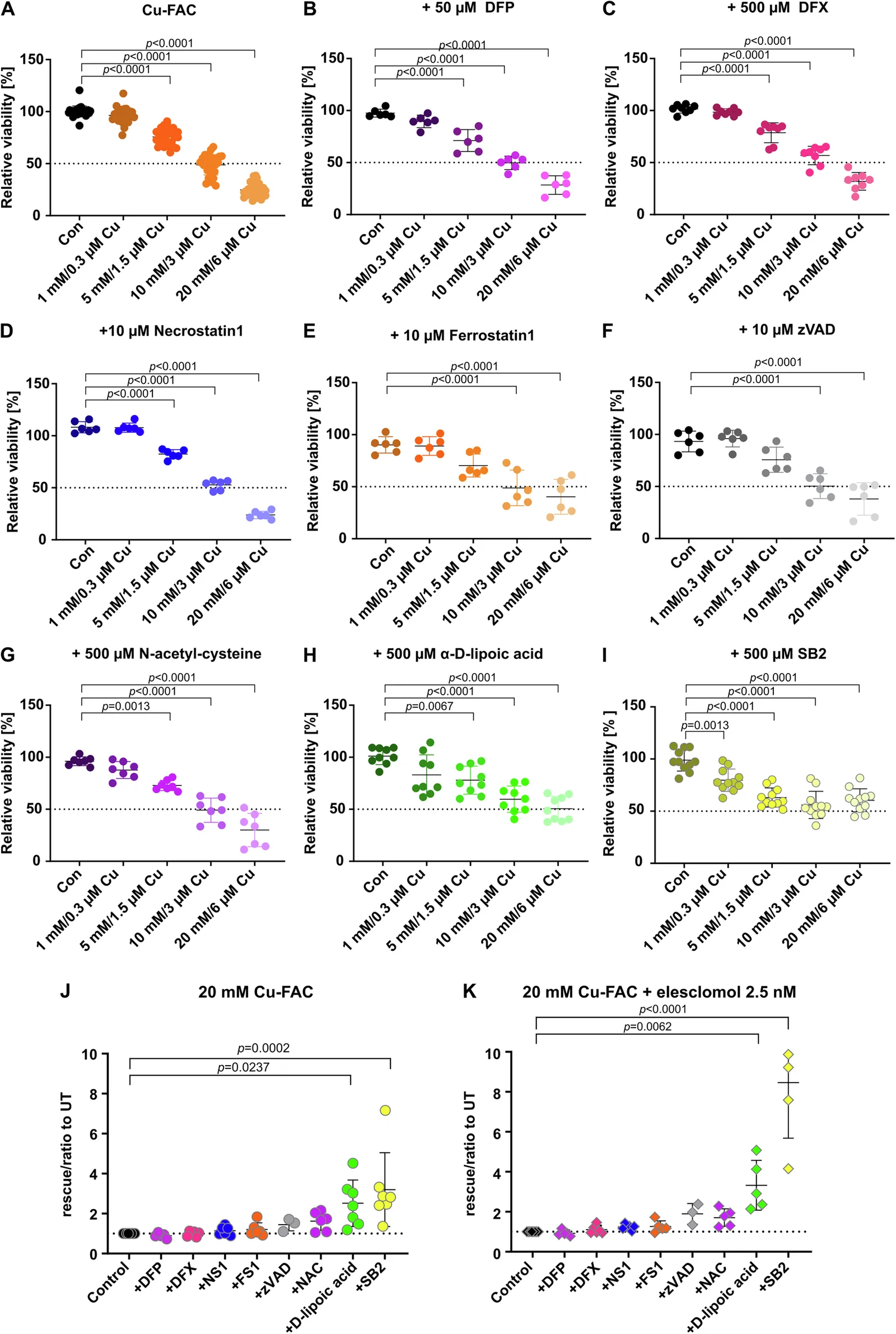
Dithiol-containing compounds rescue copper-driven iron toxicity, unaffected by apoptosis, necroptosis, or ferroptosis inhibitors. **A** CellTiter-Glo® assay of Huh7 cells treated for 24 hours with increasing concentrations of Cu-FAC. Data are expressed as percentage vs. untreated controls (*N* = 6, *n* = 2). Cell viability after 24 h FAC-Cu treatment preceded by 24 h pretreatment with: 50 µM deferiprone (DFP, **B**), 500 µM deferasirox (DFX, **C**), 10 µM necrostatin-1 (**D**), 10 µM ferrostatin-1 (**E**), 10 µM zVAD-fmk (zVAD, **F**), 500 µM N-acetylcysteine (NAC, **G**), 500 µM α-D-lipoic acid (**H**), or 500 µM SB2 (**I**) (*N* ≥ 3, *n* = 2). **J** Rescue ratio calculated as viability with compound + Cu-FAC divided by viability with Cu-FAC alone (*N* ≥ 3, *n* = 2). **K** Rescue ratio in the presence of 2.5 nM elesclomol (*N* ≥ 3). For statistical evaluation, one-way ANOVA was employed, only in comparison to the control. The horizontal line shows the mean, with the error bars representing the standard deviation.

To further investigate the mechanism of Cu-induced iron excess toxicity, we evaluated diverse preincubation countermeasures to avoid Huh7 demise (Fig. 5A–I). Here, inhibitors were used at concentrations that did not provoke cell toxicity per se, and their individual potential therapeutic effect was compared by calculating the ratio of the viability of the respective compound plus Cu-FAC divided by the viability with Cu-FAC alone (Fig. 5J, K).

Neither iron-chelating compounds (DFX and DFP [46]), nor necroptosis (necrostatin-1 [47]), nor ferroptosis (ferrostatin-1 [48]) inhibitors prevented Cu-FAC toxicity (Fig. 5), in agreement with FAC alone being non-toxic and with our supposition that this toxicity is not due to Fenton chemistry-induced oxidative stress (Fig. 5B–E, J, K). Pretreatment with the thiol-containing antioxidant N-acetylcysteine (NAC) [49] elicited a very mild but insignificant protective effect, similar to the apoptosis inhibitor zVAD-fmk [50] (Fig. 5F, G, J, K). Remarkably, a profound protection was observed, however, with the di-thiol-containing agents’ α-D-lipoic acid [51], and, most strikingly, with the high affinity copper chelator SB2 [29] (Fig. 5H–K; 5I: D-lipoic acid to control *p* = 0.0237; SB2 to control *p* = 0.0002) that was even more prominent when Cu-FAC toxicity was aggravated by the Cu-ionophore elesclomol (Fig. 5K, SFigs. 3; D-lipoic acid to control *p* = 0.0062; SB2 to control *p* < 0.0001). Thus, Cu-FAC toxicity is clearly not initiated by iron-dependent Fenton chemistry oxidative stress, as iron chelators and ferroptosis inhibitors were without protective effect, but is plausibly due to proteotoxic stress, as di-thiol-containing agents were therapeutically effective, in agreement with the observed rise in GSSG (Fig. 4G, 10 mM Cu-FAC to control *p* = 0.0229).

### Copper-driven iron toxicity targets iron-sulfur cluster proteins

**Fig. 6:**
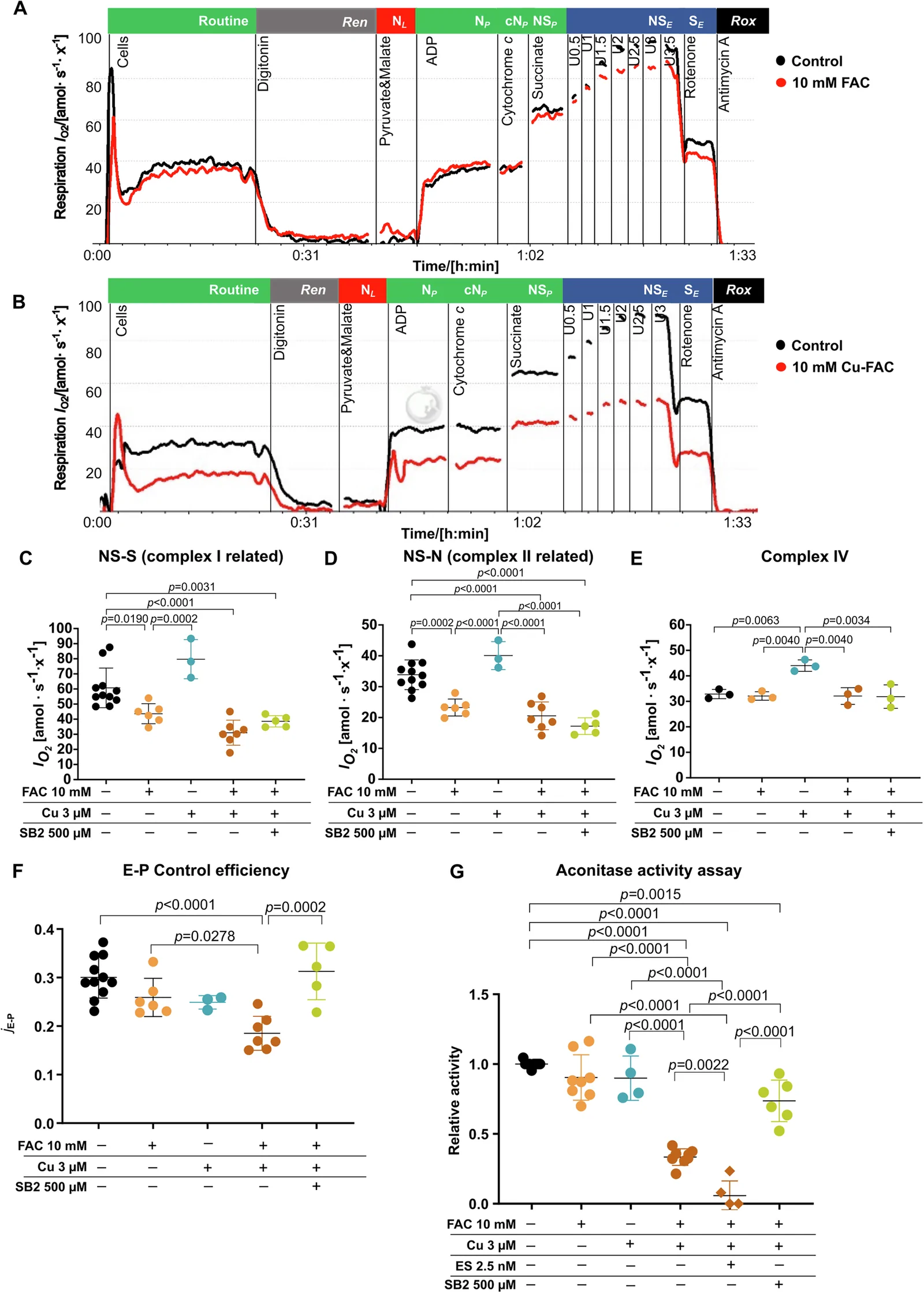
Copper-driven iron toxicity impairs iron-sulfur cluster proteins and mitochondrial respiration in Huh7 cells. **A**, **B** Representative oxygen flux traces (amol·s^−^^1^·cell^−1^) from high-resolution respirometry (SUIT-008 D025 protocol) of untreated and 10 mM FAC-treated or 10 mM Cu-FAC (Cu 3 µM) treated Huh7 cells for 24 hours. **C**, **D** Differences in NS-pathway respiratory capacities (NS_E_ - S_E_ and NS_P_ - N_P_) after 24 h treatment with FAC, Cu, Cu-FAC, or SB2 plus Cu-FAC (*N* ≥ 3, n = 1). **E** Complex IV activity measured under the same conditions (*N* ≥ 3, *n* = 1). **F** E-P Quality control parameters for respiratory measurements. (**G**) Aconitase enzymatic activity in cells treated for 24 hours with FAC, Cu-FAC, Cu-FAC plus 2.5 nM elesclomol, or SB2 pretreatment plus copper-FAC. Data are expressed relative to untreated controls (*N* ≥ 4, *n* = 1). For statistical evaluation, one-way ANOVA was employed, followed by Tukey’s post hoc test for multiple comparisons. The horizontal line shows the mean, with the error bars representing the standard deviation.

Cu-FAC toxicity was sensitively assessed by the CellTiter-Glo® assay (Fig. 3B, D) that monitors cellular ATP [39]. Consequently, we next examined mitochondrial respiration in Huh7 cells, challenged by Cu-FAC using high-resolution respirometry (Fig. 6). The delta of N-linked respiration vs. NS-linked respiration was determined, providing information about complex I-related respiration (NS-S) and complex II-related respiration (NS-N). In agreement with lower ATP production/presence as demonstrated by the CTG assay (Fig. 3D), Cu-FAC had a very strong inhibitory effect on mitochondrial respiration (Fig. 6A, B), either driven by complex I (Fig. 6C, Cu-FAC to control *p* < 0.0001) or complex II (Fig. 6D, Cu-FAC to control *p* < 0.0001), but not on complex IV (Fig. 6E). Of note, FAC alone also had a significant negative effect here (Fig. 6C FAC to control *p* = 0.0190, D, FAC to control *p* = 0.0002), although to a milder extent, but clearly arguing for a converging toxicity of FAC alone and Cu-traces on mitochondrial respiration. Indeed, when assessing mitochondrial respiratory control efficiency, calculated as the difference between maximal respiration (E) and ATP-generating respiration (P) [52], this convergence was most evident as a slight decline induced by FAC alone. This decline became significant and markedly reduced upon Cu-FAC treatment, but was prevented by copper chelation with SB2, arriving at values like those observed in untreated cells (Fig. 6F Cu-FAC + SB2 to Cu-FAC *p* = 0.0002).

Intriguingly, and maybe counterintuitive, the Cu and Fe-containing respiratory complex IV was neither affected by FAC alone nor by Cu-FAC, but respiratory complexes I and II were (Fig. 6C–E). This directed our focus on Fe-S clusters as potential targets of Cu-FAC toxicity that are not present in complex IV but are functionally essential in complexes I and II [53, 54].

We therefore assessed the activity of aconitase (Fig. 6G), a key Fe-S cluster-containing enzyme crucially involved in mitochondrial activity [50]. Strikingly, the aconitase activity alterations perfectly matched our findings on overall cellular toxicity of Cu-FAC, clearly depicting it as a prime target of this toxicity (compared to control and FAC, *p* < 0.0001). While FAC or Cu alone had a minor negative effect on its activity, Cu-FAC significantly diminished its activity, which could even be aggravated by elesclomol to fully block its activity (Cu-FAC to Cu-FAC + ES *p* = 0.0022). Copper chelation, on the other hand, by thiol-containing SB2 prevented this inhibition and thus almost reached activity levels of FAC alone (Fig. 6G Cu-FAC to Cu-FAC + SB2 *p* < 0.0001).

### Copper-driven iron toxicity targets iron-sulfur cluster synthesis

**Fig. 7:**
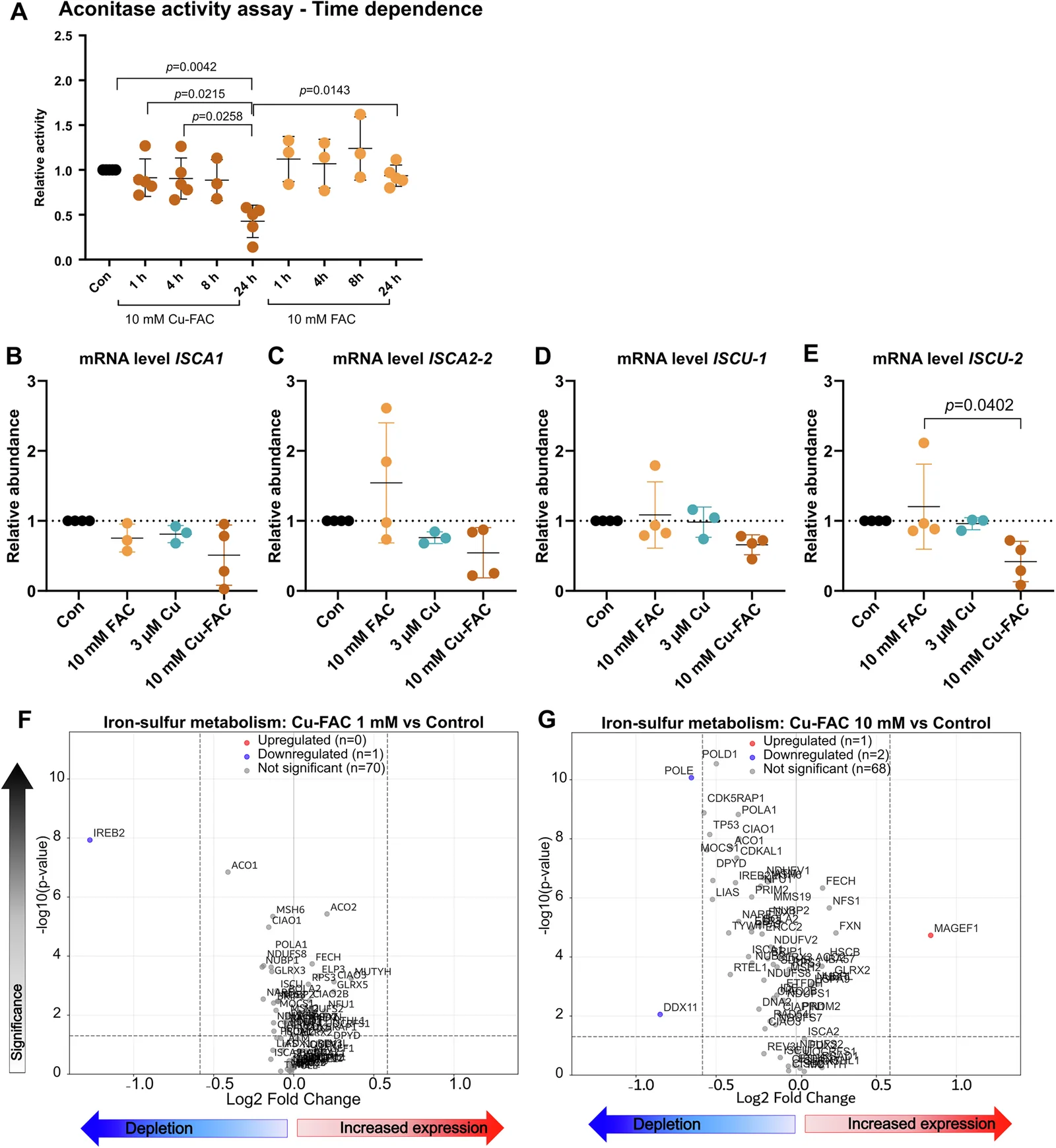
Copper-containing iron disrupts iron homeostasis by impairing iron-sulfur cluster synthesis in Huh7 cells. **A** Aconitase activity in cells treated with 10 mM FAC or Cu-FAC for 1, 4, 8, or 24 h, or left untreated. Data are expressed relative to untreated controls (*N* ≥ 3). Quantitative PCR of mRNA levels of ISCA1 (**B**), ISCA2 (**C**), ISCU-1 (**D**), and ISCU-2 (**E**) after 24 hours treatment with 10 mM FAC or Cu-FAC or 3 µM Cu-His (*N* = 3, *n* = 1) is depicted as relative abundance compared to a housekeeping gene (HPRT) and then to untreated cells. For statistical evaluation, one-way ANOVA was employed, followed by Tukey’s post hoc test for multiple comparisons. The horizontal line shows the mean, with the error bars representing the standard deviation. Changes in the proteome of Huh7 cells treated with 1 mM (**F**) or 10 mM Cu-FAC (**G**) for 24 hours, filtered for proteins involved in iron-sulfur cluster metabolism (*N* = 4) depicted as a volcano plot. Data points represent log2 fold change (x-axis) versus -log10(*p*-value) (*y*-axis). Dotted lines indicate significance thresholds (*p* < 0.05) and fold change >1.5).

As copper-initiated iron overload toxicity becomes apparent hours upon incubation (Fig. 3F), we next asked whether such delay is reflected by the impaired aconitase activity as well, which was found to be the case (Fig. 7A). Cu-FAC significantly decreased aconitase activity only after hours of incubation, while FAC alone also had a downward tendency then, although to a much lesser extent (Fig. 7A Cu-FAC 24 h to control *p* = 0.0042; Cu-FAC 24 h to FAC 24 h *p* = 0.0143). This clearly validated aconitase activity as a direct Cu-FAC target, again arguing against an immediate Fenton chemistry mechanism of toxicity, but raising the question whether this delay may be, at least in part, due to a Cu-FAC-altered Fe-S cluster biogenesis. Indeed, in Huh7 cells treated with 10 mM Cu-FAC, several Fe-S cluster assembly factors had a downward trend in mRNA and protein abundance (Fig. 7B-E, SFigs. S4A, B; ISCU-2 Cu-FAC to control *p* = 0.0402). More broadly, proteomic profiling further validated a consistent tendency towards a reduced abundance of proteins involved in Fe-S cluster metabolism, which was already evident upon FAC alone treatment, but became most apparent upon a 10 mM Cu-FAC challenge (Fig. 7F, G, SFig. S4C). It thus appears that copper-initiated iron toxicity broadly targets Fe-S cluster homeostasis, to especially apparent by reduced activities of mitochondrial complexes I and II, and aconitase (Fig. 6C, D, G).

### Copper-driven iron toxicity deteriorates mitochondria

**Fig. 8:**
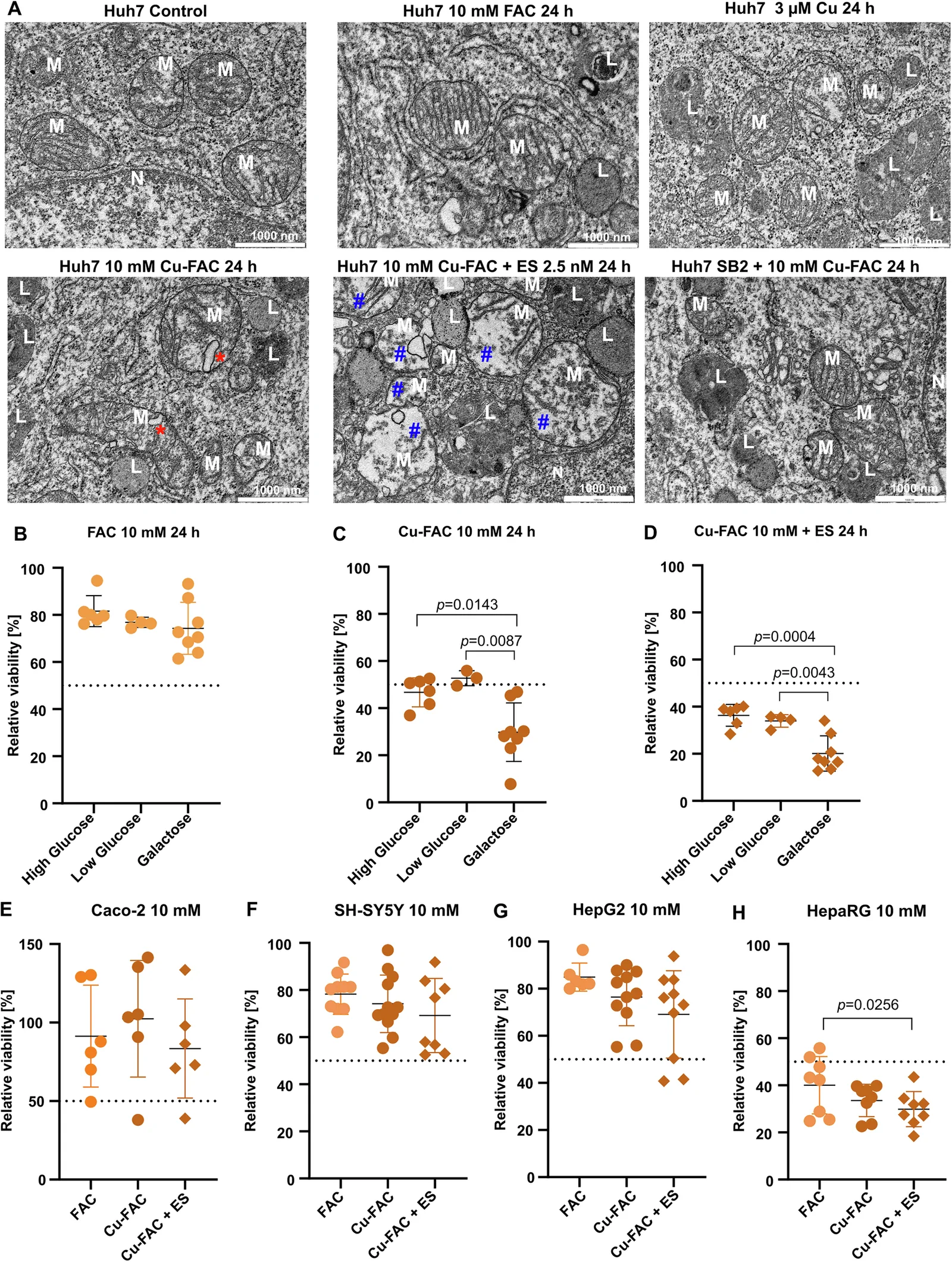
Copper-driven iron toxicity targets hepatocytes. **A** Electron micrographs depicting mitochondrial morphology under the indicated treatments, showing lysosomes (**L**) and mitochondria (**M**) with dilated cristae (*) and loss of matrix electron density (**#**). Huh7 cells were cultivated in DMEM containing 25 mM glucose (high glucose), 5.5 mM glucose (low glucose) or 5 mM galactose and treated with **B** 10 mM FAC, **C** 10 mM Cu-FAC or **D** 10 mM Cu-FAC and 2.5 nM elesclomol for 24 hours (*N* ≥ 4, *n* = 2). Caco-2 (**E**), SH-SY5Y(**F**), HepG2 (**G**), and HepaRG (**H**) cells were either treated with 10 mM FAC or Cu-FAC with or without 2.5 nM elesclomol for 24 hours (*N* ≥ 3, *n* = 2). Cell viability was assessed via CellTiter Glo. Data are given as a percentage of untreated cells. For statistical evaluation, one-way ANOVA was employed, followed by Tukey’s post hoc test for multiple comparisons. The horizontal line shows the mean, with the error bars representing the standard deviation.

Iron overload combined with copper traces or enforced copper entry by elesclomol severely impaired Huh7 bioenergetics, particularly mitochondrial respiration (Figs. 3E, F, 6). While Fe-S dependent enzymes were major targets, mitochondrial structure and function were broadly compromised (Figs. 8A, 6). Iron alone caused minimal ultrastructural changes, consistent with findings in hemochromatosis mice (Fig. 1F, G), but addition of copper triggered cristae dilation and functional loss (Figs. 8A, 6; SFig. S5). The most pronounced damage occurred upon additional elesclomol presence, causing swelling and matrix disruption characteristic of mitochondrial destruction and cell death (Fig. 8A). Notably, the copper chelator SB2 largely prevented detrimental Cu-FAC defects, restoring normal matrix density and function (Figs. 5K, 6F, G, 8A). Thus, Cu-FAC structurally impairs mitochondria, leading to energetic deficits as evidenced by the CellTiter-Glo® assay that monitors cellular ATP (Fig. 3B, D). Indeed, Cu-FAC toxicity was further exacerbated upon shifting the Huh7 cells from glucose to galactose medium, which enforces mitochondrial ATP production (Fig. 8B–D), validating mitochondria as key targets of copper-enhanced iron toxicity.

### Cuproptotic-like cell death prominently targets iron-overloaded hepatocytes

To evaluate the relevance of copper-enhanced iron toxicity beyond hemochromatosis surrogate hepatocytes (Huh7), we cross-examined further cell types. Interestingly, Caco-2 cells, surrogate intestinal cells, were largely resistant to such toxicity (Fig. 8E), and oxidative stress-sensitive neuronal surrogate cells (SH-SY5Y) were only mildly affected (Fig. 8F), comparable to hepatocyte HepG2 cells (Fig. 8G). Most relevant, however, was an encountered Cu-FAC sensitivity, again especially and significantly when enforced by elesclomol, of differentiated HepaRG cells, an immortalized hepatic cell line that closely matches primary human hepatocytes (Fig. 8H Cu-FAC to Cu-FAC + ES *p* = 0.0256). Thus, the induction of cuproptosis by elesclomol/Cu prominently targets iron-overloaded hepatocytes.

## Discussion

Hereditary hemochromatosis (HH) is characterized by excessive hepatic iron accumulation, yet its clinical penetrance remains remarkably low. Even among individuals with comparable iron burdens, disease presentation ranges from asymptomatic to severe organ injury [11, 47]. The molecular basis of this heterogeneity is poorly defined but likely reflects modifying factors that alter cellular responses to iron overload.

In this study, we report profound cell death of hemochromatosis Huh7 cells elicited by copper traces in iron overload situations, whereas such copper traces alone were harmless to these cells. With respect to potential pathological relevance, intracellular metal concentrations were benchmarked against human disease values. Patients with HH typically exhibit hepatic iron concentrations of 8-30 mg/g dry weight, representing a 5- to 100-fold increase over normal levels (0.3–1.5 mg/g) [47]. Our experimental models recapitulated this range: *Hfe* KO mice displayed a three- to four-fold hepatic iron increase (Fig. 1C), while Huh7 cells treated with 1 mM FAC accumulated ~27-fold more iron (Fig. 3G, I), comparable to severe iron overload in HH. Thus, although the maximal Huh7 iron load in this study represents a very elevated pathological scenario, it is even more surprising that no overt cell toxicity was apparent. On the contrary, and in agreement with earlier studies, we found that iron overload alone in either hemochromatosis *Hfe* KO mice or Huh7 cells comes with cellular, especially lysosomal, adaptations to balance such burden [46, 48]. Although overt cytotoxicity was not detected, excess iron caused mild bioenergetic alterations affecting mitochondrial function, e.g., as detected among others by the CellTiter-Glo® assay that monitors cellular ATP.

Intriguingly, the addition of copper, even in trace amounts, dramatically increased this bioenergetic toxicity. In one line of initial experiments, we came across this by noting that a copper-containing FAC batch did impair mitochondrial structure, whereas an essentially copper-free FAC batch did not (Fig. 8A). Indeed, such copper impurities in iron salts have very recently also been reported by others [49]. We consequently validated next that such copper amounts alone were harmless to the cells and their mitochondria but only occurred synergistically with iron excess.

Mechanistically, this enforced toxicity appeared not to be mediated by Fenton chemistry-based oxidative stress, as toxic Cu-FAC only provoked intermediate ROS resulting in significantly less lipid peroxidation compared to non-toxic FAC (Fig. 4). Instead, we detected proteotoxic stress as indicated by a decreased GSH/GSSG ratio (Fig. 4G), in addition to elevated HSP70 (Supplementary Fig. S8C). Indeed, HSP70 has been reported to be upregulated in cuproptosis (see below) to protect cellular proteins from stress-induced denaturation, misfolding, and aggregation [51, 55].

Cu-FAC proteotoxicity especially targeted the activity of the Fe-S cluster-dependent enzymes aconitase, as well as respiratory complexes I and II, possibly in consequence of reduced core Fe-S cluster biosynthetic proteins, including ISCA1, ISCA2, and ISCUs (Figs. 6, 7). As iron alone had a mild negative effect here as well, it appears that copper massively aggravated such an iron-related adverse effect. In agreement, recent evidence demonstrated that copper ions disrupt Fe-S cluster biogenesis by direct competition with iron for key protein binding sites within the assembly machinery [56]. Moreover, it has been reported that copper binding to ISCA1, ISCA2, and mitochondrial ferredoxin blocks the assembly or maturation of [4Fe-4S] clusters [56–58]. Clearly, blocking Fe-S cluster synthesis directly impairs mitochondrial respiration and can trigger cell death, even in the absence of substantial ROS or lipid peroxidation [56, 59], which is in agreement with the results of the present study.

Mitochondria emerged as a primary target of Cu-FAC toxicity. Ultrastructural analyses revealed cristae dilation, swelling, and matrix disruption following co-exposure, which paralleled the decline in respiratory activity. Moreover, enforcing mitochondrial respiration by culturing cells in galactose medium, thereby restricting glycolytic ATP synthesis, substantially increased sensitivity to Cu-FAC, further validating mitochondria as the critical site of such vulnerability.

Regarding the Cu-FAC-elicited mode of cell death, inhibitor experiments with zVAD-fmk, necrostatin-1, or ferrostatin-1 failed to rescue Huh7 cells (Fig. 5), questioning apoptosis, necroptosis, or ferroptosis. In addition, the latter cell death mode was further ruled out as iron chelation had no protective effect (Fig. 5), and proteomic analysis did not reveal downregulation of GPX4 or upregulation of ACSL4, two characteristic markers of ferroptosis (Supplementary Fig. S7).

Rather, we find a cuproptotic or cuproptosis-like mode of Cu-FAC elicited cell death, comprising most of its typical features (i) mitochondria being targeted; (ii) proteotoxic stress as evidenced by a fading GSH/GSSH ratio (Fig. 4G–I) and HSP70 induction (Supplementary Fig. S8C), a typical hallmark of cuproptosis; (iii) a drastic aggravation of toxicity by the prime cuproptosis eliciting ionophor elesclomol (Figs. 3, 6 and 8); (iv) effective avoidance of Cu-FAC toxicity by high affinity copper- (but not iron-) chelation next to D-lipoic acid (Fig. 5), the originally described cuproptosis inhibitor; (v) a pronounced loss of Fe-S cluster enzyme activity, again a cuproptosis hallmark, most prominently for aconitase that exactly matches overall Cu-FAC cellular toxicity; (vi) a significant accumulation of DLAT aggregates upon Cu-FAC treatment (Supplementary Fig. S8A, B).

A further hallmark of cuproptosis is its reported “dependence” on FDX1, as mitochondria-residing FDX1 may specifically reduce Cu(II)-elesclomol to mitochondriotoxic Cu(I) [60, 61]. Consequently, reduced FDX1 levels may mitigate cuproptosis. Indeed, we tested for such amelioration by siRNA-specific knockdown of FDX1 (Supplementary Fig. 9A, B). While 10 µM Cu alone did not cause any toxicity (SFig. 9C), the mixture of 10 µM Cu plus 2.5 nM elesclomol was devastating, and in agreement with the earlier reports [55, 62, 63], a significant but limited protection resulted from FDX1 depletion (SFig. 9D). In contrast, such limited protection was not observed upon challenge with Cu-FAC, neither with nor without elesclomol (SFig. 9 E/F). This latter finding agrees well with our proposed mode of toxicity, in which only copper traces are needed to elicit excess iron toxicity. In this situation, an additional liberation of some extra Cu - still bound to elesclomol - by FDX1, or its avoidance, is obviously of minor/no effect. Clearly, while elesclomol presence eradicated the cells treated by Cu-FAC (SFig. 9F), this effect was rather due to the elesclomol-facilitated cellular Cu entry per se, and not due to its targeted, FDX1-dependent mitochondrial release. In fact, already in the seminal report that coined the term cuproptosis, this cell death mode was observed in copper overload mice despite lower FDX1 levels [55]. Furthermore, another study in hepatocytes reported that FDX1 knockdown does not necessarily confer resistance to copper-elesclomol-induced toxicity, whereas FDX1 overexpression enhances sensitivity [64]. Thus, differing FDX1 levels appear as a potential modulator for cuproptosis, especially in the context of liberating further toxic copper from elesclomol; however, its depletion does not avoid Cu-FAC toxicity. Nevertheless, due to the presence of multiple other hallmarks of cuproptosis, together with the absence of features of other cell death pathways, we suggest that Cu-FAC induces a cuproptosis-like cell death in Huh7 cells.

Among the cancer-derived models, Huh7 and HepG2 hepatoma cells showed clear Cu-FAC toxicity, while Caco-2 cells appeared less sensitive, likely because their high‑glucose culture conditions allow them to compensate for mitochondrial impairment by shifting toward glycolysis. SH-SY5Y neuroblastoma cells, despite also being cultured in high glucose, exhibited similar sensitivity to HepG2 cells, which may be explained by their low glutathione content that limits proteotoxicity avoidance [65]. Importantly, primary-like differentiated HepaRG cells, which display mature hepatocyte characteristics and intact mitochondrial capacity, had an even greater susceptibility to Cu-FAC than the cancer-derived hepatocytes (Fig. 8). The pronounced vulnerability of these differentiated hepatocytes, together with the strong metabolic dependence revealed in the galactose medium experiments (Fig. 8B–D), suggests that cells with active mitochondrial metabolism are particularly prone to Cu-FAC toxicity.

This cellular vulnerability provides a mechanistic framework to interpret existing in vivo evidence of copper-iron dysregulation. Copper-dependent ferroxidases (hephaestin, ceruloplasmin) critically control intestinal iron absorption and hepatic iron efflux, establishing tight metabolic interdependence between these metals [22, 66]. Indeed, dietary iron overload consistently induces systemic copper deficiency in both humans and animal models [19, 20, 67], potentially reflecting iron-mediated inhibition of copper absorption or possibly active hepatic protection against dual metal excess [19]. Critically, copper supplementation during dietary iron overload caused marked hepatomegaly in rats, accompanied by exacerbated iron accumulation, which might be a direct demonstration of copper-enhanced iron liver toxicity [19]. Thus, while copper-enhanced iron toxicity must be further investigated in vivo in future studies, it is nevertheless most apparent from this study that copper may be one of the most devastating modifiers of iron-overload disease that, importantly, can be alleviated by high-affinity copper chelation.

Two major limitations of this study should be acknowledged. Firstly, the employed high iron concentrations appear to be supraphysiological but match relative elevations in pathological hemochromatosis. Secondly, in vivo studies will be essential to unravel under which pathological situations such copper-elicited iron excess toxicity becomes detrimental.

## Conclusions

Hepatic iron load is largely balanced in hemochromatosis mice and surrogate hepatocytes. In contrast, trace copper elicits and massively exacerbates iron toxicity by interfering with iron-sulfur cluster-containing proteins and mitochondrial bioenergetics. This potent mechanism of cytotoxicity can be substantially attenuated by high-affinity copper chelation. Our findings highlight not only the pathological significance of copper-enhanced iron overload but also propose aconitase activity as mechanistically grounded biomarker to monitor cuproptotic processes in future investigations.

Collectively, these results identify copper as a decisive modifier of iron toxicity, thereby arguing for a detrimental dual-metal interplay as a plausible explanation for the variable penetrance of hemochromatosis, where subtle differences in hepatic copper content could determine whether iron accumulation remains compensated or transitions into cytotoxicity.

## Material and Methods

Chemicals were purchased from Sigma-Aldrich (Taufkirchen, Germany), Merck (Darmstadt, Germany), Serva Electrophoresis GmbH (Heidelberg, Germany), Thermo Fisher Scientific (Massachusetts, USA), Roche (Penzberg, Germany) and VWR International GmbH (Ismaning, Germany). Glutaraldehyde, osmium tetroxide and Uranyless were purchased from Science Services GmbH (Munich, Germany) and lead citrate from Leica Biosystems (Wetzlar, Germany). Methanobactin (SB2) was isolated from *Methylocystis sp*. SB2 as previously described [68, 69].

ADP, malate, N-acetyl-cysteine, pyruvate, SB2, and succinate were dissolved in deionized H_2_O, pH checked, and adjusted to pH 7 if necessary. Alpha-D-lipoic acid, BODIPY C11, CM-H_2_DCFDA, deferiprone, desferasirox, digitonin, elesclomol, ferrostatin-1, Hoechst 33342, LysoTracker™ Deep Red, necrostatin-1, and zVAD-fmk were dissolved in DMSO. Antimycin A, rotenone, and CCCP were dissolved in ethanol. Different stocks of FAC, one with undetectable low/absent copper (<100 nM), the other one containing higher levels of copper (30 µM in 100 mM FAC stock, molar ratio of 1:333) and FAC (“Cu-free”) supplemented with CuCl_2_ (molar ratio 1:3333, comparable to Cu levels in Cu-contaminated FAC) were also dissolved in deionized H_2_O, pH checked and adjusted to pH 7, furthermore metal content was routinely validated by ICP-OES (Arcos II, SPECTRO Analytical Instruments, Kleve, Germany). All solutions were aliquoted and stored at −20 °C, except for pyruvate, which was prepared freshly before each measurement, and FAC, Cu-FAC, N-acetyl-cysteine, and SB2, which were stored at 4 °C.

### Animals

C57BL/6 J *Hfe*^-/-^ mice were obtained from the laboratory of Prof. Maja Vujić Spasić at Ulm University, Germany [24]. Animals were maintained under the Guidelines for the Care and Use of Laboratory Animals of Helmholtz Munich, following the EU Directive 2010/63/EU.

All mice were housed under specific pathogen-free (SPF) conditions in individually ventilated cages with *ad libitum* access to a standard chow diet (1314; 191.02 mg Fe/kg, 13.89 mg Cu/kg; Altromin Spezialfutter GmbH, Seelenkamp, Germany) and distilled water. All mice showed no signs of pain or disease at the time of investigation.

### Histological examination

Mouse liver tissues were fixed for 48 hours at room temperature in 4% paraformaldehyde and embedded in paraffin. Tissue samples were cut into 4 µm-thick sections and stained with hematoxylin and eosin (H&E) for analysis. Embedding and H&E staining were performed by the Institute of Pathology, TUM School of Medicine and Health at the Technical University of Munich. Paraffin-embedded liver sections were stained for iron using Perls’ blue and counterstained using nuclear fast red according to standard protocols by the Institute of Anatomy, Ludwig-Maximilians-Universität, Munich. Stained sections were imaged with a light microscope (Axio Imager.M2, ZEISS, Oberkochen, Germany) equipped with a 40x objective (Plan-Apochromat 40x, ZEISS, Germany) and operated with Stereo Investigator software (MBF Bioscience, Williston, VT, USA). Images were analyzed using an image browser (Biolucida Viewer, MBF Bioscience) and a raster graphics editor (Affinity Photo 2, Serif Europe Ltd., Nottingham, UK), where only contrast and brightness adjustments were made without altering the appearance of the original materials.

### TBARS Determination

Lipid peroxidation in liver tissue was assessed by the Institute of Comparative Molecular Endocrinology at Ulm University, by measuring thiobarbituric acid-reactive substances (TBARS) following the original protocol [70]. In brief, frozen liver tissues, with an average weight of 0.05 g, were homogenized in cold 1.15% KCl, centrifuged, and the supernatant collected. Thereafter, 0.8% TBA (thiobarbituric acid), prepared in 20% acetic acid (pH 3.5) and 10% sodium dodecyl sulfate (SDS), was added to the liver homogenate, and the mix was incubated for 1 hour at 95 °C for adduct formation between the TBA and lipid peroxides. Samples were then cooled down and incubated for 5 minutes in ice prior to the addition of n-butanol/pyrimidine (15:1; v/v), the mixture was centrifuged at 4000 rpm/10 min at room temperature, and the optical density was read at 532 nm. TBARS/malondialdehyde (MDA) levels were calculated using a standard curve of 1,1,3,3-tetramethoxypropane (TMP), and the concentration was expressed as nmoles/mg protein.

### Transmission Electron microscopy

For transmission electron microscopy, a piece of fresh liver tissue or Huh7 cell pellets (3·10^5^ cells per sample) was immediately fixed in 2.5% glutaraldehyde, post-fixed with 1% osmium tetroxide, dehydrated with acetone, embedded in epoxy resin (Epon 812 according to Dalton), cut in ultrathin sections (50 nm) and analyzed in a transmission electron microscope (Jeol 1200 EXII [60 kW], equipped with a KeenViewII digital camera) [71–73].

### Cell culture

Huh7 (human hepatoma) cells supplied by Sekisui XenoTech LLC were obtained from the JCRB Cell Bank (Tokyo, Japan) and cultured in Dulbecco’s Modified Eagle Medium (DMEM; Thermo Fisher Scientific, Massachusetts, USA) containing 5.5 mM glucose, supplemented with 10% fetal calf serum (FCS; Bio&Sell, Feucht, Germany) and 1% penicillin/streptomycin (Thermo Fisher Scientific, Massachusetts, USA).

Caco-2 (human epithelial colorectal adenocarcinoma) cells (ECACC® 86010202, UK, provided by Sigma-Aldrich, Taufkirchen, Germany) were cultured in DMEM containing 25 mM glucose (Thermo Fisher Scientific, Massachusetts, USA), supplemented with 10% fetal bovine serum (FBS; Bio&Sell, Germany), 1% penicillin/streptomycin, and 1% non-essential amino acids (NEAA; Thermo Fisher Scientific, Massachusetts, USA).

SH-SY5Y cells (ATCC, Wesel, Germany) were cultured Medium DMEM (Thermo Fisher Scientific, Massachusetts, USA) containing 25 mM glucose, supplemented with 10% FCS (Biochrom, Germany), and 1% penicillin/streptomycin (Thermo Fisher Scientific, Massachusetts, USA).

Hepatocellular carcinoma HepG2 cells (ATCC, HB-8065) were maintained at 37 °C in 5% CO_2_ and cultured in DMEM (Thermo Fisher Scientific, Massachusetts, USA) containing 5.5 mM glucose, supplemented with 10% FCS (Bio&Sell, Feucht, Germany) and 1% penicillin/streptomycin (Thermo Fisher Scientific, Massachusetts, USA).

HepaRG cells were cultured and differentiated by LHP in Williams’ E medium supplemented with 10% FCS, 1% penicillin/streptomycin, 1% L-glutamine, 0.023 IU/mL insulin, 4.7 µg/mL hydrocortisone, and 80 µg/mL gentamicin. All supplements were obtained from Thermo Fisher Scientific (Massachusetts, USA), except for insulin (Sanofi Aventis, Frankfurt am Main, Germany), hydrocortisone (Pfizer Inc., New York, USA), and gentamicin (Teva Pharmaceutical Industries Ltd., Petah Tikva, Israel). For differentiation, cells were cultured in the same medium supplemented with 1.8% DMSO for four weeks, with medium changes performed once per week. All cells were maintained at 37 °C in a humidified atmosphere with 5% CO_2_ and routinely tested for mycoplasma contamination using the mycoplasma test kit (AppliChem GmbH, Darmstadt, Germany). Furthermore, all electron micrographs of the cells were regularly checked for mycoplasma contamination.

### Knockdown of FDX1 using siRNA

FDX1-targeting siRNA, control siRNA, and the corresponding transfection reagent were obtained from Dharmacon™ (Dharmacon Inc., Lafayette, CO, USA) and used as ON-TARGETplus siRNA SMARTpools according to the manufacturer’s instructions. Cells were seeded in 6-well plates at a density of 2·10^5^ cells per well and allowed to adhere for 4 hours prior to transfection.

For transfection, 5 pmol of siRNA was mixed with the transfection reagent in serum- and antibiotic-free DMEM and incubated at room temperature for 20 min. An equal volume of DMEM supplemented with 10% FBS and 1% penicillin-streptomycin was then added to the mixture. The culture medium was aspirated from the cells, and the transfection mixture was applied. Cells were incubated for 36–48 h, after which knockdown efficiency was confirmed by immunoblotting.

### Fluorescence staining with Lysotracker™ Deep Red

Huh7 cells were plated at 2·10^4^ per well in 8-well chamber slides (Ibidi, Gräfelfing, Germany) and allowed to grow overnight. Thereupon, cells were treated for 1, 6, or 24 hours with 10 mM ferric ammonium citrate (FAC) or incubated with DMEM only. After the respective incubation time, cellular nuclei were counterstained with Hoechst 33342; Lysotracker Deep Red 633 (Molecular Probes) was used as a marker for lysosomes, following the manufacturer’s instructions. Fluorescence imaging was performed with a Nikon Eclipse Ti-S microscope (Nikon Metrology, Leuven, Belgium) at 600x magnification. Lysosomal morphology and mean fluorescence intensity per cell were analyzed using ImageJ ROI Manager.

### CMH_2_CDFA ROS production assay

Huh7 cells were seeded at a density of 1·10^4^ cells/well in 96-well plates. After 24 hours, increasing concentrations of FAC +/− copper (Cu-FAC: 100 mM FAC stock supplemented with 30 µM Cu, 3333:1) or DMEM only and 10 µM CMH_2_DCFDA (in PBS) were added to the corresponding wells. The fluorescent signal was immediately measured using a ClarioStar plate reader (BMG Labtech, Ortenberg, Germany). The plate was then incubated at 37 °C and 5% CO_2_ for 6 hours, with ROS production measured every hour.

### BODIPY C11 lipid peroxidation measurement

Huh7 cells were seeded at a density of 1·10^4^ cells/well in 96-well plates. The next day, 1 or 10 mM FAC or Cu-FAC (with 0.3 or 3 µM Cu, respectively) or DMEM only was added. After 1, 4, 6, or 24 hours' incubation, the medium was removed, cells were washed with PBS, and incubated with medium containing 5 µM of BODIPY 581/591 C11 for 30 min in the incubator. After washing, the plate was analyzed in the ClarioStar plate reader (BMG Labtech, Ortenberg, Germany), measuring the fluorescence at 480 nm and 610 nm. Fluorescence-activated cell sorting (FACS) analysis was also performed as confirmation of this assay. Huh7 cells were seeded at a density of 5·10^5^ cells/well in 6-well plates. The next day, 10 mM FAC or Cu-FAC (with 3 µM Cu, respectively) or DMEM only was added. After 24 hour incubation, 5 µM of BODIPY 581/591 C11 was added to the cells and further incubated for 1 hour at 37 °C. Thereupon, the cells were harvested, transferred in PBS + 5% FCS and the cells analyzed in the BD Accuri™ C6 plus flow cytometer (BD, Benton Dickinson GmbH, Heidelberg, Germany) and the measurement evaluated using FlowJo™ v10 software (FlowJo LLC, BD, Benton Dickinson GmbH, Heidelberg, Germany). Lipid peroxidation was evaluated by a fluorescence emission shift from 610 to 480 nm.

### GSH assay

GSH/GSSG levels were determined according to Rahman et al. [74] with slight modifications. Cell pellets from 1·10^6^ cells were resuspended in 100 µL isolation buffer (300 mM sucrose, 5 mM TES, 0.2 mM EGTA) and mixed with equal volumes of 10% metaphosphoric acid and sonicated three times for 10 sec with 65% amplitude on ice. Subsequently, samples were neutralized by addition of triethanolamine. Total GSH content was assessed in the presence of reaction buffer (100 mM potassium phosphate, 0.3 mM NADPH, 20 units of glutathione reductase, pH 7.4) and 0.2 mM 5,5’-dithiobis-(2-nitrobenzoic acid) (DTNB). The formation of 5′-thio-2-nitrobenzoic acid (TNB) was recorded at 412 nm via a ClarioStar plate reader (BMG Labtech, Ortenberg, Germany). For GSSG determination, the samples were previously processed by masking GSH with 9% 2-vinylpyridine and thereupon measured as described before.

### Cytotoxicity assays: CellTiter-Glo® and Neutral Red uptake assay

Huh7, HepG2, Caco-2, and SH-SY5Y cells were seeded at a density of 1·10^4^ cells/well and HepaRG at a density of 3.4·10^4^ cells/well in 96-well plates and in some cases, directly incubated with chelators/inhibitors. After 24 hours, increasing concentrations of FAC or Cu-FAC were added +/- elesclomol. The cells were incubated for another 24 hours, and cell viability was determined via the CellTiter-Glo® assay (Promega, Madison, USA) according to the manufacturer’s instructions or the Neutral Red uptake assay [75] in a ClarioStar plate reader (BMG-Labtech, Ortenberg, Germany).

### Aconitase assay

Huh7 cells (1.5·10^6^ cells) were incubated for 24 hours with 10 mM FAC or Cu-FAC (3 µM Cu) in the absence or presence of 500 µM Methanobactin SB2 (SB2) (pretreated for 24 hours) or 2.5 nM elesclomol (co-treatment), respectively. Afterwards, cells were trypsinized and counted. Cell viability was determined by Erythrosin B staining. 2·10^6^ cells were centrifuged and stored frozen at -80 °C. For the assay, 2·10^6^ cell pellet was resuspended in 220 µL TRIS 50 mM pH 7.4 buffer (containing protease inhibitor cOmplete™ EDTA-free). After three freeze-thaw cycles, the sample was centrifuged at 800 g for 10 min and supernatant transferred into a new vial. The aconitase activity was determined as previously described [76]. Briefly, to 150 µL assay buffer (50 mM TRIS, 60 mM Na-Citrate, 1 mM MnCl_2_, 0.2 mM NADP^+^ and 0.2 g/L isocitrate dehydrogenase, pH 7.4) 50 µL cell lysate was added, and NADPH production was followed for 60 min at 340 nm in the ClarioStar plate reader at 37 °C, in 1 min intervals. Aconitase activity was calculated from its linear slopes.

### High-resolution respirometry

Huh7 cells were pretreated for 24 hour with DMEM alone or containing 10 mM FAC, 3 µM Cu, or 10 mM Cu-FAC (3 µM Cu) +/− 500 µM SB2. Oxygen consumption was assessed by high-resolution respirometry (HRR) using the Oxygraph-2k and DatLab 7.0 (Oroboros Instruments GmbH, Innsbruck, Austria) as described previously [77]. Per chamber, 0.8·10^6^ living cells were supplied in 2 mL of MiR05 buffer (0.5 mM EGTA, 3 mM MgCl_2_, 60 mM lactobionic acid, 20 mM taurine, 10 mM KH_2_PO_4_, 20 mM HEPES, 110 mM sucrose, 1 g/L albumin, pH 7.1), and routine respiration was measured. Addition of 5 µg/mL digitonin permeabilized the plasma membrane of the cells, and respiration depended on residual endogenous substrates inside the mitochondria. Pyruvate and malate were added (2 & 5 mM, respectively), enabling measurement of the leak state in the N-pathway. Thereupon, 2 mM ADP was added and oxidative phosphorylation (OXPHOS) was recorded. Succinate (10 mM) addition induced NS-linked OXPHOS respiration, and stepwise titration of CCCP (3-4 µM) allowed the determination of the maximum electron transfer (ET) and thereby the maximum capacity of the electron transport system. Inhibition of NADH-dehydrogenase (complex I) by 0.5 µM rotenone addition enabled measurement of the S-linked ET respiration. Addition of 2.5 µM antimycin A inhibited ubiquinol cytochrome *c* reductase (complex III), and thus non-mitochondrial oxygen consumption (ROX) was measured.

The assessment of cytochrome *c* oxidase (COX or complex IV) activity started with a 20 min incubation period, during which the chambers remained open after the addition of 2 mM ascorbate and 0.5 mM TMPD. After chamber closure, oxygen consumption was monitored for 5 min. To determine the chemical background oxygen flux, 200 mM azide, a COX inhibitor, was then added. The COX activity was calculated by subtracting the background flux (after azide addition) from the signal under ascorbate and TMPD.

### Immunoblotting

For immunoblotting, cell lysates were prepared using cell extraction buffer (Thermo Fisher Scientific, Waltham, USA), supplemented with cOmplete protease inhibitor cocktail (Roche, Penzberg, Germany) according to the manufacturer’s instructions. The samples were denatured at 95 °C for 5 min with Laemmli buffer containing 5% β-mercaptoethanol before separation via SDS-PAGE. Following electrophoresis, the samples were transferred to an PVDF membrane (0.45 µm, Merck, Darmstadt, Germany), activated previously with methanol incubation, and probed overnight at 4 °C with antibodies against DLAT (1:1000, Thermo Fisher Scientific, Massachusetts, USA, cat.# MA5-35723), FDX1 (1:1000, Abcam, Cambridge, United Kingdom), Ferritin (1:1000, Abcam, Cambridge, United Kingdom, cat.# ab65080), NCOA4 (1:500, Thermo Fisher Scientific, Massachusetts, USA, cat.# PA5-39311), ISCA1 (1:1000, Thermo Fisher Scientific, Massachusetts, USA, cat.# PA5-60121) ISCA2 (1:1000, Abcam, Cambridge, United Kingdom, cat.# ab235776), and GAPDH as loading control (1:1000, Cell Signaling, Leiden, Netherlands, cat.# 2118). The membranes were incubated with horse-radish peroxidase-conjugated secondary antibodies. After the addition of LumiGLO® substrate (Cell Signaling, Leiden, Netherlands), chemiluminescence was detected with a Fusion FX (Vilber Lourmat Deutschland GmbH, Eberhardzell, Germany), the program FusionCapt Advanced was used to quantify the band intensities.

### Metal content determination

Liver metal content from *Hfe* knockout mice was partially provided by BA. Liver homogenate was prepared by rinsing livers with ice-cold 0.9% NaCl, placing them in isolation buffer (300 mM sucrose, 5 mM TES, 0.2 mM EGTA, 0.1% (w/v) BSA, pH 7.2), and preparing a 20% (w/v) homogenate using a Teflon-glass homogenizer (5–10 strokes, 800 rpm, B. Braun, Melsungen, Germany). The homogenate was diluted with 65% HNO_3_ to a final concentration of 33% HNO_3_ and digested using a Discover SP-D clinical microwave system (CEM, Kamp-Lintfort, Germany).

For cellular metal content, 1.5 × 10^6^ cells were seeded and incubated for 24 hours with 5 µM Cu-His, 10 mM FAC, or Cu-FAC, with or without 500 µM SB2 in DMEM with 10% FCS. After treatment, cells were trypsinized, counted, and viability was assessed by erythrosine B staining. Viable cells (1–2 × 10^6^) were collected, centrifuged, washed with PBS, and pellets were stored at −80 °C for at least 1 hour before analysis. For metal determination, pellets were digested in 33% HNO_3_ for 48 hours at 95 °C. Iron, copper, and zinc were quantified from all samples by ICP-OES (Arcos II, SPECTRO Analytical Instruments, Kleve, Germany).

### RNA isolation and quantitative PCR

RNA was isolated from 1-2·10^6^ Huh7 cells using the RNeasy Plus Mini kit (QIAGEN, Hilden, Germany) according to the manufacturer’s instructions. RNA purity and concentration were assessed via a NanoDrop spectrophotometer (Thermo Fisher Scientific, Massachusetts, USA). The QuantiTec Reverse Transcription Kit (QIAGEN, Hilden, Germany) was utilized to reverse-transcribe 1–10 µg RNA into cDNA. Thereafter, quantitative PCR was run on a LightCycler® 480 System (Roche Diagnostics, Penzberg, Germany) using Takyon NoRox SYBR Mmx dTTP blue 7.5 (Eurogentec, Seraing, Belgium). Relative expression was calculated using 2^-ΔΔCt^ method with HPRT as the housekeeping gene and Huh7 untreated cells as control. Primer sequences for ISCA1, ISCA2-1, ISCA2-2, ISCU-1, ISCU-2 and HPRT are listed in Table 1.

**Table 1:** Primers used for quantitative PCR.

Primer	Forward 5’ - 3’	Reverse 3’ - 5’
ISCA1	GATGTCGGCTTCCTTAGTCC	CATTACAGCCCCTGGTTCGG
ISCA2-1	AGTTATCAACCCCGACGACA	GCCTTGCTGTGCTTGAGGAT
ISCA2-2	AGAGAGCGGTCACTCCCTG	TACCCTGGACGCAACTGTCT
ISCU-1	GGAATTGGGGGTCACAAATGG	GTCTTGTCAAGGGACCCCAC
ISCU-2	AACATTTGGCTGTGGTTCCG	ATCTTCAGCCAGCATGGAGC
Ferroportin (FPN)	CCCTGGGCCCCTTTTCTTT	TCAGGTAGTCGGCCAAGGAT
Ferritin (FTH)	TCGGGGTTTCCTGCTTCAAC	CCACATCATCGCGGTCAAAG
HPRT	TCAGGCAGTATAATCCAAAGATGGT	AGTCTGGCTTATATCCAACACTTCG

### Proteome analysis

1.5·10^6^ cells were incubated for 24 hourrs with 1 or 10 mM FAC or Cu-FAC in DMEM. Afterwards, cells were trypsinized and counted. Cell viability was determined by Erythrosin B staining. 1-2·10^6^ cells were pelleted and washed once with PBS. The protein concentration was determined by the Bradford assay, and a total of 50 µg protein per replicate was stored at -80 °C. These samples were then further prepared by CvT using the iST kit (PreOmics, Munich, Germany) for MS measurement.

#### MS measurement

MS data were acquired in DIA-PASEF mode on a timsTOF HT mass spectrometer (Bruker, Bremen, Germany). Equal amounts of peptides were loaded onto the online-coupled VanquishNeo (Thermo Fisher Scientific, Dreieich, Germany) HPLC system. A Nano-Trap column was used (300 µm inner diameter (ID) × 5 mm), packed with Acclaim PepMap100 C18, 5 µm, 100 Å from LC Packings, Sunnyvale, CA, USA, before separation by reversed-phase chromatography (Aurora 75 µm ID × 250 mm, 1.8 µm from Ionoptics, Fitzroy, Australia) at 40 °C. Peptides were eluted from the column at 250 nL/min using increasing ACN concentration in 0.1% formic acid from 3 to 40% over a 45 min gradient. The DIA-PASEF method covered a mass range from 300 to 1250 m/z and a mobility range from 0.65 to 1.35 1/ko. Precursor peptides were isolated with 34 equal windows. Collision energy for 0.6 1/ko was set to 20 and for 1.6 1/ko to 59. Estimated cycle time was 1.91 s.

#### Data processing

DIA files were processed with Spectronaut (Version 19, Biognosys) as direct DIA analysis against a SwissProt human database (Release 2020_02, 20432 sequences) using BSG factory settings for Pulsar search. For DIA analysis, default settings were applied. For quantification, precursor filtering was set on Q-value, with cross-run normalization. Imputation was set to run-wise. Quantity MS level was set to MS2; Quantity type was area, and protein quantification is based on the summed peptide intensities. Protein-group-specific peptides were allowed for quantification. The mass spectrometry proteomics data have been deposited to the ProteomeXchange Consortium via the PRIDE [78] partner repository with the dataset identifier PXD070837.

#### Protein mapping and pathway analysis

The quantified data were further preprocessed by JG in Python using specified dependencies including pandas (≥2.3.0), scikit-learn (1.6), matplotlib (3.9), and the Reactome API. First, protein identifiers were extracted and data cleaned for API compatibility. Annotation was achieved by submitting protein identifiers to retrieve relevant biological pathways, with gene-to-pathway mapping accomplished via Reactome’s GMT file, supplemented by API queries for missing data. The analysis focused on iron-related biological processes using semantic keyword search patterns (“iron|ferr|heme”) in the descriptive columns of the proteome data (‘GO Cellular Component’, ‘GO Molecular Function’, ‘GO Biological Process’). Results were visualized through volcano plots for all underlying pathways and per individual pathway.

### Miscellaneous

Cellular protein levels were determined by the Bradford assay [79]. Cells were counted using a LUNA-II™ Automated Cell Counter (Logos Biosystems, Anyang, South Korea).

### Statistical analysis

Data are presented as mean ± standard deviation, with *N* representing the number of biological replicates and *n* representing the number of technical replicates. Two groups were compared using the unpaired t-test (two-tailed). For multiple group comparisons involving a single variable, one-way ANOVA or Kruskal-Wallis was employed, with Tukey’s post hoc test for multiple comparisons. Analyses were done using GraphPad Prism 7 (GraphPad Software, Inc., San Diego, CA, USA).

## Supplementary information

Supplementary Material

Original Data: Western blot

## Data Availability

The datasets generated during and/or analyzed during the current study are available from the corresponding author on reasonable request.
