# Supplementary material for: Cuproptosis in iron overload hepatocytes
Source: Cell Death Dis. 2026 Sep 19;17(1):816. doi: 10.1038/s41419-026-09275-y (PMC13589748; doi:10.1038/s41419-026-09275-y)
Supplement: Supplementary file 1 [file 41419_2026_9275_MOESM1_ESM.docx]

Supplementary Material

*Supplementary figure S1. Changes in iron metabolism. (A)* *Quantification of mean LysoTracker™ fluorescence from (B) using the ImageJ ROI Manager (N = 3). (B) Exemplary Immunoblot analysis of Huh7 cells treated with 10 mM FAC for 1, 4, 7, or 24 h, or untreated (Con), probed for NCOA4 and GAPDH (loading control); quantified with FusionCapt Advance (N ≥ 3). (C) Exemplary Immunoblot analysis after 24 h treatment with increasing FAC concentrations or untreated (Con), probing ferritin and GAPDH. Densitometric quantification performed using FusionCapt Advance software (N ≥ 3). For statistical evaluation one-way ANOVA was employed, followed by Tukey's post hoc test for multiple comparisons. The horizontal line shows the mean, with the error bars representing the standard deviation.*

*Supplementary figure S2. Copper has a limited co-toxic effect on iron. (A) Cell viability measured by CellTiter-Glo® after 24 h treatment with increasing concentrations of copper with or without the presence of 2.5 nM elesclomol (ES). Data are expressed as percentage of untreated controls (N = 6). (B) Viability after 24 h treatment with 0.5 mM FAC with increasing concentration of copper with or without the presence of 2.5 nM elesclomol (ES) (N = 4). (C) Viability after 24 h treatment with 1 mM FAC with increasing concentration of copper with or without the presence of 2.5 nM elesclomol (ES). Data are expressed as percentage of untreated controls (N ≥ 4). (D) Direct comparison of cell viability of Huh7 cells treated with different concentration of FAC with or without copper addition (10 µM) without (left) or in the presence of elesclomol (2.5 nM) (N ≥ 4).* *For statistical evaluation one-way ANOVA was employed, followed by Tukey's post hoc test for multiple comparisons. The horizontal line shows the mean, with the error bars representing the standard deviation.*

*Supplementary figure S3. Thiol-containing compounds protect against copper-induced iron toxicity in the presence of elesclomol (ES), whereas apoptosis, necroptosis, and ferroptosis inhibitors do not. (A) CellTiter-Glo® assay of Huh7 cells exposed for 24 h to increasing concentrations of copper-supplemented FAC in the presence of elesclomol (ES). Results are shown as percentage of untreated controls (N = 6). (B–I) (B–I) Cell viability after 24 h Cu-FAC treatment in the presence of 2.5 nM ES preceded by 24 h pretreatment with: 50 µM deferiprone (DFP, B), 500 µM deferasirox (DFX, C), 10 µM necrostatin-1 (D), 10 µM ferrostatin-1 (E), 10 µM zVAD-fmk (F), 500 µM N-acetylcysteine (NAC, G), 500 µM α-D-lipoic acid (H), or 500 µM SB2 (I) (N ≥ 3, n = 2 each). For statistical evaluation one-way ANOVA was employed, followed by Tukey's post hoc test for multiple comparisons. The horizontal line shows the mean, with the error bars representing the standard deviation.*

*Supplementary figure S4. Immunoblot analysis of ISCA1 (A), ISCA2 (B), and GAPDH protein levels in treated cells with quantification by FusionCapt Advance (N = 3).* *For statistical evaluation one-way ANOVA was employed, followed by Tukey's post hoc test for multiple comparisons. The horizontal line shows the mean, with the error bars representing the standard deviation. (C) Changes in the proteome of Huh7 cells treated with 1 mM or 10 mM FAC for 24 h, filtered for proteins involved in iron-sulfur cluster metabolism (N = 4) depicted as volcano plot.* *Data points represent log2 fold change (x-axis) versus -log10(p-value) (y-axis). Dotted lines indicate significance thresholds (p<0.05 and fold change>1.5).*

Supplementary figure S5: Electron micrographs of Huh7 cells, treated for 24 h with 10 mM FAC, 10 mM Cu-FAC, Cu-FAC plus 2.5 nM elesclomol, or 500 µM SB2 plus Cu-FAC or left untreated (control).

Supplementary figure S6: Cell viability and intracellular metal content of treated Huh7 cells. (A) Cell viability after 24 h treatment with 10 mM FAC, 10 mM Cu-FAC, Cu-FAC plus 2.5 nM elesclomol, or 500 µM SB2 plus Cu-FAC of left untreated (Con). Data are expressed as percentage of untreated controls (N ≥ 4). (B, C) Intracellular iron (B) and copper (C) content measured by ICP-OES from cells treated as in (A). For statistical evaluation one-way ANOVA was employed, followed by Tukey's post hoc test for multiple comparisons. The horizontal line shows the mean, with the error bars representing the standard deviation.

Supplementary figure S7: Markers of ferroptosis. Changes in the proteome of Huh7 cells treated with 10 mM FAC or Cu-FAC for 24 h or left untreated (Control), filtered for proteins known as activators of inhibitors of ferroptosis (N = 4) depicted as table. Highlighted lines indicate significance thresholds (p<0.05 and fold change>1.5 or <0.7).

**C**

Supplementary figure S8: Markers of cuproptosis. (A) Immunoblot analysis of Huh7 cells treated with 10 mM FAC or Cu-FAC for 24 h, or untreated (Con), probed for DLAT and GAPDH (loading control); quantified with FusionCapt Advance (N ≥ 3). (B) Densitometric quantification performed using FusionCapt Advance software. The horizontal line shows the mean, with the error bars representing the standard deviation. (C) Changes in the proteome of Huh7 cells treated with 10 mM FAC or Cu-FAC for 24 h or left untreated (Control), filtered for proteins known as markers for cuproptosis (N = 4) depicted as table. Highlighted lines indicate significance thresholds (p<0.05 and fold change>1.5 or <0.7).

Supplementary figure S9: Dependence on FDX1. (A) Immunoblot analysis of Huh7 cells treated with non-targeting siRNA (Con) or siRNA targeting FDX1 for 72 h, probed for FDX1 and GAPDH (loading control); quantified with FusionCapt Advance (N ≥ 3). (B) Densitometric quantification performed using FusionCapt Advance software. The horizontal line shows the mean, with the error bars representing the standard deviation. (C-F) Viability of Huh7 cells treated for 48 h with siRNA, followed by co-treatment for 24 h with 10 µM Cu (C), 10 µM Cu + 2.5 nM elesclomol, (D), 10 mM Cu-FAC (E) or 10 mM Cu-FAC+2.5 nM elesclomol. Data are expressed as percentage of untreated controls (N = 4). An unpaired t-test was employed for statistical evaluation.

*Fluorescence staining of Huh7:*

*Control (each row represents one biological replicate)*

10 mM 1 h:

10 mM 6 h:

10 mM 24 h
