# Supplementary material for: Cuproptosis in iron overload hepatocytes
Source: Cell Death Dis. 2026 Sep 19;17(1):816. doi: 10.1038/s41419-026-09275-y (PMC13589748; doi:10.1038/s41419-026-09275-y)
Supplement: Supplementary file 2 [file 41419_2026_9275_MOESM2_ESM.docx]

*Immunoblot replicates NCOA4:*

*Immunoblot replicates ferritin:*

*Immunoblot replicates ISCA1 and ISCA2:*

*Immunoblot DLAT:*

*Immunoblot FDX1:*
